# Anaerobic riboflavin degradation by human gut *Lachnospiraceae*

**DOI:** 10.1128/jb.00108-26

**Published:** 2026-07-20

**Authors:** Christian J. Quiles Pérez, Alexandria Olzak, Aminata Fofana, Kamal Deep, Calissa Carlisle, Emily Bradley, Kathryn Kananen, Larry Beaver, Connor Skaggs, Justin A. North, Patrick H. Bradley

**Affiliations:** 1 Department of Microbiology, The Ohio State University 2647, Columbus, Ohio, USA; 2 Infectious Diseases Institute, The Ohio State University 2647, Columbus, Ohio, USA; 3 Center of Microbiome Science, The Ohio State University 2647, Columbus, Ohio, USA; University of Massachusetts Chan Medical School, Worcester, Massachusetts, USA

**Keywords:** gut microbiome, *Lachnospiraceae*, cellular redox status, anaerobic catabolic pathways, riboflavin, metabolism, lumichrome, hydroxyethylflavin

## Abstract

Vitamins mediate a web of cross-feeding interactions in the human gut. Many gram-positive gut microbes, in particular, are predicted to be vitamin auxotrophs. Previous studies of these microbes, however, have tended to use rich media, precluding controlled perturbations of low-abundance nutrients. We tested the ability of diverse *Lachnospiraceae*, the most common gram-positive bacteria in the gut, to grow on a chemically defined medium. Even though this medium contained riboflavin, we found that predicted riboflavin auxotrophs grew poorly, including the bile metabolizer *Clostridium scindens*. High-dose riboflavin supplementation enhanced growth, but also revealed that, surprisingly, *C. scindens* catabolizes riboflavin into lumichrome, making it the first reported anaerobe to do so. The only previously described catabolic pathway for riboflavin requires oxygen and has no homologs in *C. scindens*. In high-dose riboflavin, a single gene neighborhood with an aldolase, oxidoreductases, and a riboflavin kinase/adenylyltransferase was upregulated, suggesting an alternative anaerobic degradation or overflow pathway. Similar neighborhoods were detected in several other *Lachnospiraceae*, including *Faecalicatena fissicatena*, the only other anaerobe reported to degrade riboflavin. Reanalysis of published metabolomic data showed that, *in vivo*, both riboflavin and lumichrome were more abundant in colonized (vs germ-free) mouse ceca, and that, *in vitro*, *Lachnospiraceae* isolates depleted riboflavin while certain gram-negative isolates overproduced it. These results demonstrate that a member of the *Lachnospiraceae* can anaerobically convert an essential B vitamin into lumichrome, a molecule recently shown to have anti-inflammatory properties. Vitamin catabolism may both structure cross-feeding interactions in the gut and affect host health.

**IMPORTANCE:**

*Lachnospiraceae*, the most prevalent human gut gram-positive bacteria, produce many health-relevant metabolites, but are genetically intractable and often grown in rich medium, complicating physiological studies. Unexpectedly, through comparative experiments in a chemically defined medium, we identify the first anaerobe that can catabolize riboflavin to lumichrome and show that it induces a specific gene neighborhood while doing so, suggesting a novel pathway. Variants of this neighborhood are conserved in a handful of *Lachnospiraceae*, including the only other anaerobe reported to degrade riboflavin (to hydroxyethylflavin). These results potentially explain decades-old observations implicating gut microbes in riboflavin catabolism. Furthermore, riboflavin catabolites have recently been shown to inhibit host mucosal-associated invariant T (MAIT) cell activation, suggesting an additional mechanism by which commensal *Lachnospiraceae* may dampen inflammation.

## INTRODUCTION

Gut microbial metabolism drives many of its links to host health: certain microbial metabolites can feed host cells (1), regulate immune responses (2), and inhibit pathogens (3, 4), while others may, for example, worsen renal disease (5) or predispose the host to atherosclerosis (6, 7). Modulating this metabolic activity therefore offers a route toward novel gut-targeted therapeutics, whether via small-molecule inhibitors of microbial pathways (8) or altering the nutritional environment, for example, by providing prebiotics (9). Prebiotics modulate the dietary molecules available to gut microbes; however, the gut microbiome also extensively metabolizes host molecules (e.g., mucin, urea, and lactate) (10–12) as well as nutrients from other members of the community via cross-feeding (13).

Cross-feeding between members of the gut microbiome may be particularly relevant for vitamins (13). Vitamins are usually required in small amounts, and the host efficiently absorbs many vitamins in the proximal small intestine (14). Thus, in the absence of large-dose supplementation that overwhelms this machinery, microbial sources of vitamins may be especially important *in vivo*. In synthetic communities where auxotrophies of various types were introduced by gene deletion, vitamin auxotrophy specifically was found to impose low costs on growth rate while leading to stable cross-feeding relationships (15). Many gut microbes are predicted, based on genome sequences, to be vitamin auxotrophs (16), and co-culture experiments have confirmed that auxotrophs for quinones (17) or B vitamins (18) can indeed be fed by gut prototrophs.

Vitamin availability may also affect which specific pathways gut microbes can perform and, therefore, which specific products are formed. For example, pseudovitamin B_12_ provision by *E. hallii* shifts carbon metabolism in the mucus degrader *A. muciniphila* to favor propionate over succinate (19), while supplementation with very high-dose riboflavin has been shown to shift gut community metabolism toward the production of butyrate (20) without major alterations to community structure. Both propionate and butyrate are health-associated short-chain fatty acids (SCFAs) that modulate a range of host phenotypes, ranging from immunity to metabolism (21), illustrating the potential impact of vitamin-dependent metabolic remodeling.

As redox balance is one of the most important determinants of metabolic routes in gut anaerobes (22), vitamins that form redox-active cofactors, such as nicotinate and flavins, are good candidates for influencing gut microbial metabolism. Flavins play especially critical roles for anaerobes. Flavodoxins often substitute for ferredoxins in iron-limited environments and are therefore critical for activating certain radical enzymes, as well as providing electrons to hydrogenase. Hydrogen itself is an important redox “currency metabolite” in the gut, whose concentration can itself affect metabolic flux (23). Flavoproteins also transfer electrons to alternative acceptors, such as urocanate and fumarate (24). Yet another possible role for flavins is serving as an extracellular electron shuttle, as demonstrated in *Shewanella* (25), *Listeria* (26), and potentially the gut commensal *Faecalibacterium prausnitzii* (27) (a member of the Clostridia, though not the *Lachnospiraceae*), which has been reported to use soluble riboflavin to reduce oxygen “at a distance,” similarly to how *P. aeruginosa* in biofilms is thought to use the structurally similar phenazines (28, 29).

With some exceptions, vitamins are thought to be mainly long-lived molecules that are extensively recycled, explaining why they are typically required only at low concentrations. However, vitamins may also be transformed by microbes. One type of transformation involves converting vitamins into forms that may be more or less accessible by particular taxa, as seen with, for example, vitamin B_12_ (30, 31). Cofactors containing vitamins may also be susceptible to metabolic damage through side or spontaneous reactions (32). Perhaps counterintuitively, microbes may also actively degrade vitamins, and potentially even use them as additional carbon sources if they are available in high enough concentrations, as seen with nicotinate fermentation in *E. barkeri* (33) and riboflavin catabolism in *M. maritypicum* (34) and *D. riboflavina* (35). Breakdown products of riboflavin have also previously been observed in the mammalian gut and in urine, with gut microbes implicated in their production (36–38).

Despite this, the role of vitamin degradation or catabolism in the gut microbial food web has received relatively little attention. One potential reason is that the mechanisms for such degradative pathways have not yet been elucidated, making it difficult to predict this activity from (meta)genomics data. The aerobic riboflavin degradation pathway, for instance, was only worked out in the late 2010s (34, 35), even though this activity was initially observed as early as 1944 (39). Furthermore, as a key step of this pathway requires oxygen (34, 35), there is currently no explanation for how anaerobic microbes might perform a similar activity.

In the present study, we focus on the *Lachnospiraceae*, which are the most common gram-positive family in the human gut (40) and are predicted to be auxotrophic for several B vitamins (16, 18). As these microbes produce a variety of health-relevant molecules (3, 4, 41, 42), we were motivated to investigate what growth factors they require and how nutrient availability may regulate the production of these key metabolic products. Chemically defined media are powerful tools for answering questions like these (43), as they allow precise perturbations of even low-abundance nutrients. However, while chemically defined media have been developed for certain species of the *Lachnospiraceae* (18, 41), most isolates are still typically grown in rich medium. *Lachnospiraceae* also unfortunately remain largely genetically intractable, further limiting our ability to investigate metabolic regulation. To address these limitations, we therefore took a comparative approach (44), using natural variation to link differences in growth to genetic variation. Furthermore, our experiments use a fully chemically defined medium, which we modeled on previous successful work in *C. scindens* (41) and other *Lachnospiraceae* (18).

## RESULTS

### The ability to synthesize riboflavin *de novo* explains variation in *Lachnospiraceae* growth on a chemically defined medium (DM)

**Fig 1:**
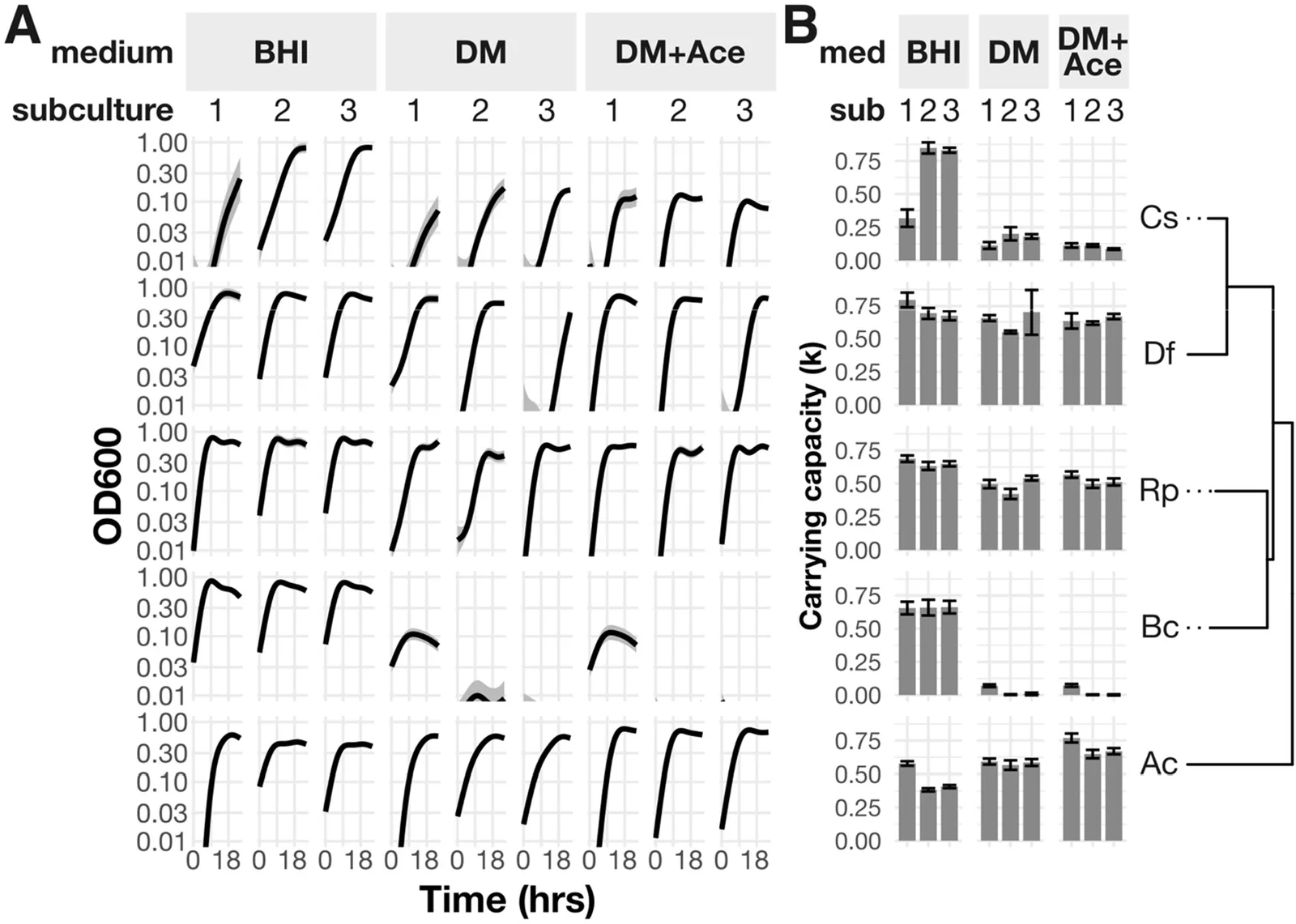
Growth of *Lachnospiraceae* isolates in rich and defined media. (**A**) Growth curves in brain heart infusion (BHI, biological *n* = 4), defined medium with fructose as the carbon source (DM, *n* = 3), and defined medium with both fructose and acetate (DM + Ace, *n* = 3). Each group had two to four technical replicates per biological replicate. The lines show the mean from a generalized additive model fit, and the shaded areas show the 95% CI. Subcultures are 20-fold dilutions into fresh media. (**B**) Estimated carrying capacity (*K*) based on a logistic curve fit. Columns are as in panel **A** (“med,” medium; “sub,” subculture). Error bars represent the estimated 95% CI from a generalized additive model. The tree shows the phylogenetic relationship between species. Species abbreviations are as follows: Cs, *Clostridium scindens*; Df, *Dorea formicigenerans*; Rp, *Robinsoniella peoriensis*; Bc, *Blautia coccoides*; Ac, *Anaerostipes caccae*.

We grew five diverse *Lachnospiraceae* in two variants of a chemically defined medium: one had fructose as a sole carbon source (DM), and one was additionally supplemented with acetate (DM + Ace). To test for sustained growth, we measured optical density across three successive subcultures. *Robinsoniella peoriensis*, *Anaerostipes caccae*, and *Dorea formicigenerans* consistently exhibited robust growth on DM, reaching a stable maximum OD similar to what they reached on the rich medium BHI + CHK (brain heart infusion with L-cysteine, hemin, and vitamin K; Fig. 1A and B). In contrast, *Clostridium scindens* grew only a very small amount in each subculture (maximum OD of approximately 0.2), and *Blautia coccoides* did not grow at all in subcultures 2 and 3. Adding acetate slightly increased the growth rate of *D. formicigenerans* and *C. scindens*, but without changing the pattern we observed in maximum OD across species.

**Fig 2:**
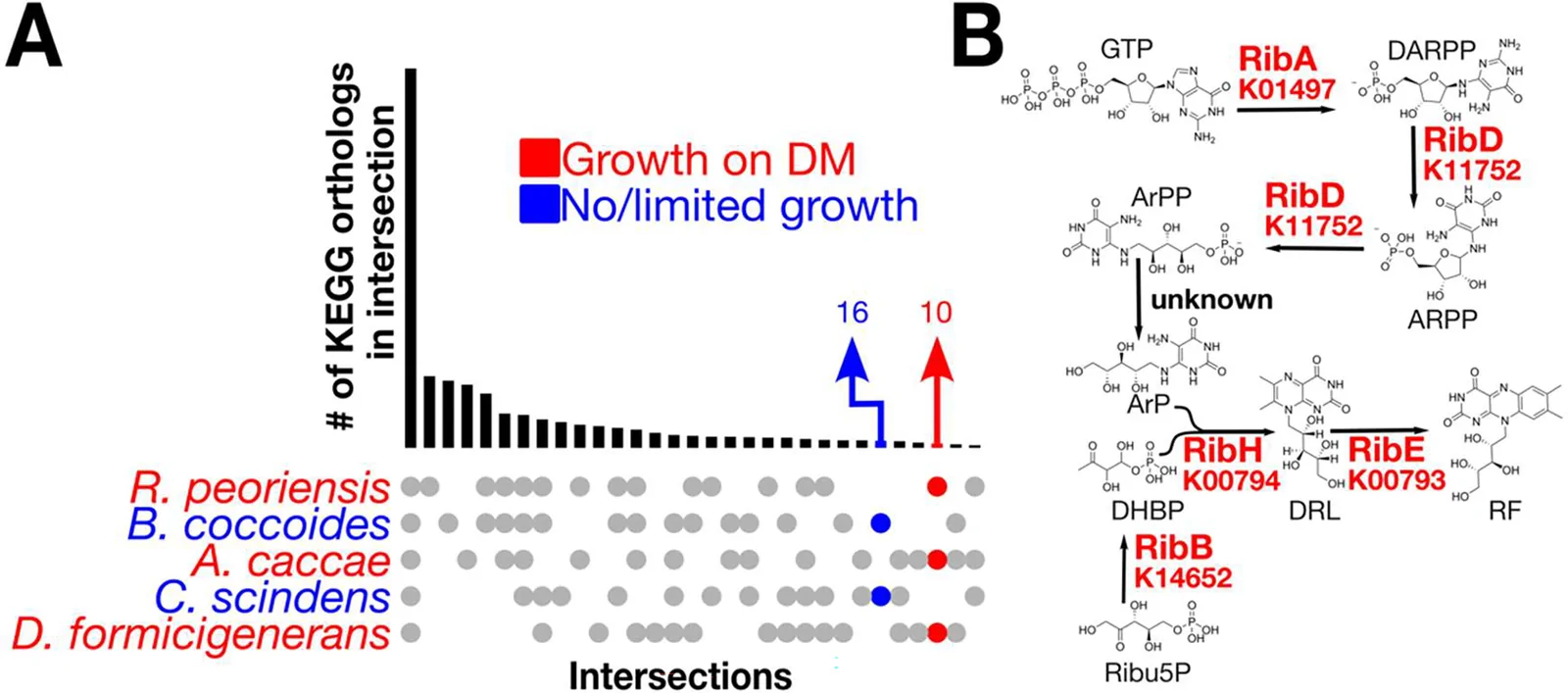
The *de novo* riboflavin biosynthesis pathway is associated with the ability to grow on DM. (**A**) UpSet plot showing the number of KEGG orthologs (KOs, black bars) that are specific to subsets of all five genomes (“intersections”; filled gray circles indicate which genomes are in the subset). Taxa that grew well on DM (red) shared 10 KOs (listed in Table S2), while taxa that did not grow well (blue) shared 16 KOs (listed in Table S3). (**B**) Diagram showing the *de novo* riboflavin biosynthesis pathway. All five KOs were present in only the strains that grew well (red). DARPP, 2,5-diamino-6-(1-D-ribosylamino)pyrimidinone phosphate; ARPP, 5-amino-6-(1-D-ribosylamino)pyrimidinone phosphate; ArPP, 5-amino-6-(1-D-ribitylamino)pyrimidinone phosphate; ArP, 5-amino-6-(1-D-ribitylamino)pyrimidinone; Ribu5P, ribulose-5-phosphate; DHBP, 3,4-dihydroxy-2-butanone 4-phosphate; DRL, 6,7-dimethyl-8-ribityllumazine; RF, riboflavin.

We set out to explain these differences based on gene content. This required us to first sequence the *R. peoriensis* type strain, which at that time did not have a full genome sequence. Using a combination of long- and short-read sequencing (see Materials and Methods), we were able to obtain a genome with only two scaffolds. Next, we annotated genomes for all five species using a consistent pipeline based on anvi’o, which we previously found had higher sensitivity to detect KEGG Ortholog families (KOfams) in taxa such as the *Lachnospiraceae* (45). We found that only 10 KOfams were present in *R. peoriensis*, *A. caccae*, and *D. formicigenerans*, but not *C. scindens* and *B. coccoides* (Fig. 2A), and of these, five belonged to a single pathway: *de novo* riboflavin biosynthesis (Fig. 2B and full set in Table S2 and Table S3).

These results pointed to riboflavin auxotrophy as a potential explanation. Indeed, *C. scindens* ATCC 35704 is already known to require riboflavin to grow (41). However, the DM we used did contain riboflavin at 0.13 µM, a concentration that was previously shown to permit growth for three other riboflavin auxotrophs from the gut (e.g., *R. inulinivorans*) (18).

Because flavins play such important roles in anaerobic metabolism, one possibility is that *C. scindens* might simply need much higher quantities of riboflavin. As mentioned, flavodoxin plays an important role in activating radical enzymes and in providing high-energy electrons to hydrogenase. *C. scindens* is a prolific producer of hydrogen (41), and both *C. scindens* and *B. coccoides* encode hydrogenase maturation cofactors absent from the other three strains we tested (Table S2 and Table S3). *C. scindens*, in particular, is also known to use flavoproteins to reduce bile acids and steroids (46, 47). Finally, flavins (including, unusually, riboflavin itself) are also components of Rnf/Nqr-like systems (48), which are present in *C. scindens*. If *C. scindens* and/or *B. coccoides* simply required larger amounts of riboflavin to grow well, then riboflavin auxotrophy could still explain the pattern of growth we observed.

### Riboflavin supplementation strongly enhances *C. scindens* growth

**Fig 3:**
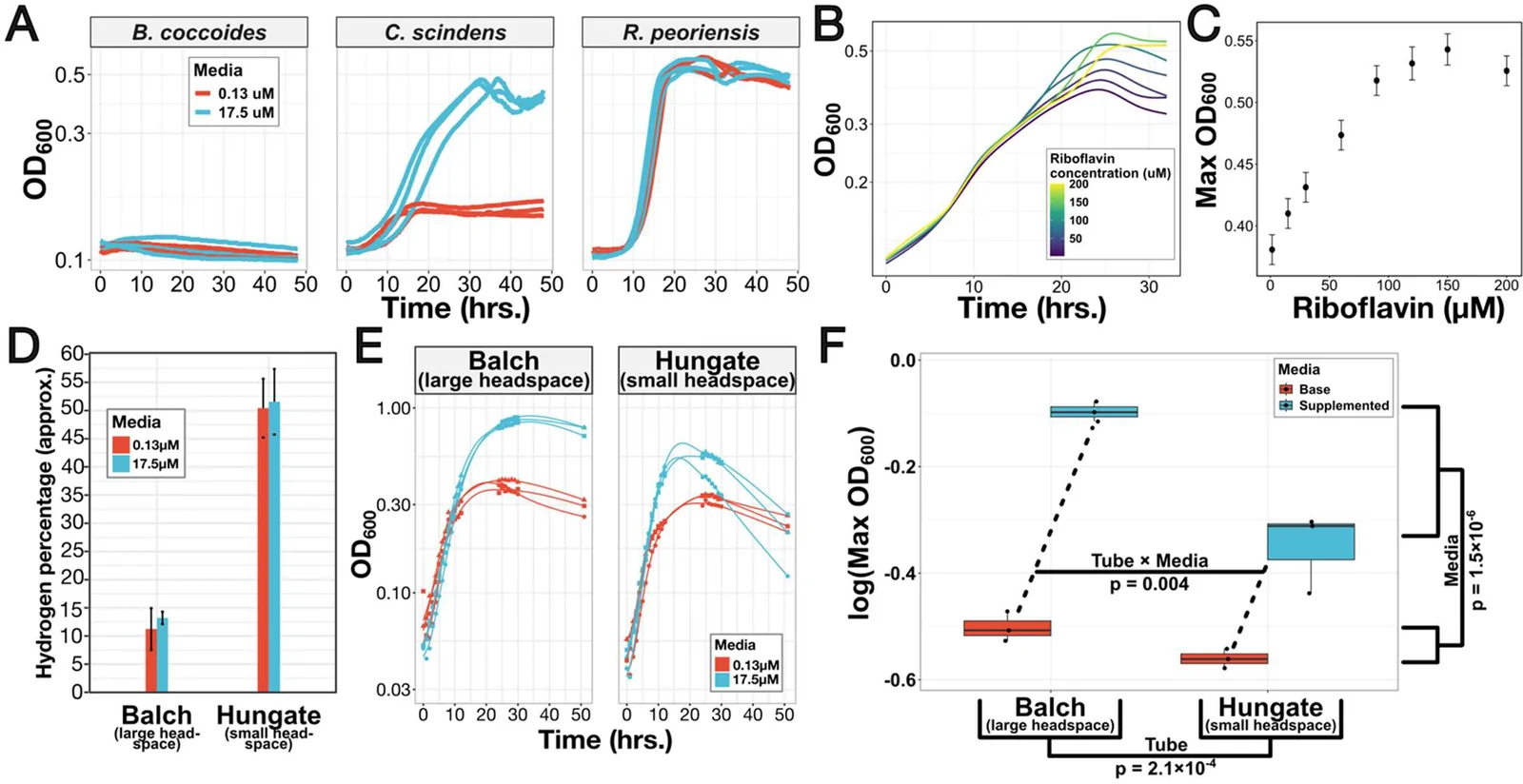
High-dose riboflavin enhances growth of *C. scindens* by a mechanism other than relief of hydrogen inhibition. (**A**) Growth curves of *B. coccoides*, *C. scindens*, and *R. peoriensis* on DM in the presence of either 0.13 µM (red) or 17.5 µM (teal) riboflavin. Each line represents one biological replicate (*n* = 3 total) and is the average of *n* = 2 technical replicates. (**B**) Growth curves of *C. scindens* on DM supplemented with a range of riboflavin concentrations (colors). Each line is the average of three biological replicates each with one to two technical replicates. (**C**) Maximum OD_600_ of *C. scindens* at different riboflavin concentrations. Error bars represent 95% CIs obtained from a linear model. (**D**) Hydrogen concentrations in Balch and Hungate tubes at 51 h growth (*n* = 3 biological replicates; error bars are 1 × SE), estimated from gas chromatography. Note that percentages are estimates because the very high H_2_ concentrations were all above the concentrations we used in our calibration curve. (**E**) Growth curves and (**F**) maximum OD_600_ of *C. scindens* grown on 0.13 µM (red) and 17.5 µM riboflavin (teal), in either Balch tubes with large headspaces (left) or Hungate tubes with small headspaces (right). *P* values are from a two-way ANOVA on the log maximum OD_600_.

We first tested the hypothesis that the concentration of riboflavin we used in our DM was limiting for *C. scindens*. Indeed, when supplemented with 17.5 µM riboflavin, *C. scindens* peaked at an OD approximately double what it reached on 0.13 µM (Fig. 3A). Furthermore, additional riboflavin continued to enhance *C. scindens* growth in a dose-dependent manner, producing significant increases in peak OD up to at least 90 µM (Fig. 3B and C). Riboflavin supplementation did not affect the growth of *B. coccoides* or *R. peoriensis* (Fig. 3A).

One potential reason we did not see strong growth with 0.13 µM riboflavin could be low or inefficient transport across the membrane. This is observed in, for example, *E. coli* riboflavin auxotrophs, which lack a dedicated transporter (49, 50) and require hundreds of micromolar riboflavin to grow. If a transporter is heterologously expressed in those strains, they then require only micromolar amounts to grow to a high OD (51). However, we found genes encoding homologs of the RibU riboflavin transporter (52) (KOfam K23675) in all five strains that we tested and actually observed a second RibU paralog in both *B. coccoides* and *C. scindens*. In *L. lactis*, where RibU was discovered, the *ribU* gene is regulated by an upstream FMN riboswitch (53) (Rfam RF00050), a gene regulatory element that acts as a terminator when bound to intracellular FMN (54). Consistent with a role in riboflavin transport, RefSeq (55) annotations for both *C. scindens* and *B. coccoides* also included an FMN riboswitch element upstream of each copy of *ribU*. This strongly suggests the presence of functional riboflavin transporters.

### Riboflavin does not enhance *C. scindens* growth by relieving hydrogen inhibition

Even with supplementation to 17.5 µM, the maximum density of *C. scindens* we observed was still much lower than that obtained previously on a very similar defined medium containing 1.7 µM riboflavin, which was actually designed for this exact strain (41). One potential explanation was hydrogen inhibition. We initially cultured *C. scindens* in 96-well plates inside an anaerobic chamber, in which the gas atmosphere contained 10% CO_2_ and between 2 and 3% H_2_. In contrast, the experiments in which *C. scindens* grew best used glass Balch tubes containing a defined gas atmosphere of 100% CO_2_ (38)*. C. scindens* is known to produce hydrogen itself (41), and hydrogenase can be easily product-inhibited. Furthermore, while hydrogen has low solubility in water, cultures in a 96-well plate would have much higher surface-to-volume ratios than Balch tube cultures, and so we would expect more exposure to atmospheric H_2_ during plate growth inside the chamber. Hydrogenase inhibition could potentially prevent flavins from being re-oxidized (e.g., because flavodoxin is its electron donor in low-iron conditions). We therefore set out to test whether riboflavin still enhanced *C. scindens* growth in low hydrogen conditions.

To do so, we grew *C. scindens* in our DM with 0.13 µM and 17.5 µM riboflavin in glass anaerobic culture tubes without shaking, using a 100% CO_2_ atmosphere (see Materials and Methods). Furthermore, we used a consistent 10 mL culture volume in two different sizes of tubes: Hungate tubes and Balch tubes. This allowed us to control the headspace, which was approximately 3 mL in Hungate tubes (“small headspace”) and 17 mL in Balch tubes (“large headspace”). As expected, hydrogen accumulated much more in the small headspace tubes (Fig. 3D), and the peak OD was significantly higher in the large headspace tubes (linear model *P* = 2.1 × 10^−4^), consistent with relief of hydrogen inhibition (Fig. 3E and F). The small headspace cultures also showed a drop in OD after hitting the maximum, possibly as a result of cell death or a stress-induced change in cell morphology. Despite this, riboflavin supplementation increased the peak OD in both headspaces (*P* = 1.5 × 10^−6^). Furthermore, riboflavin enhanced growth even more in the large headspace tubes, where hydrogen was much lower (interaction *P* = 0.004, Fig. 3F). Therefore, we conclude that hydrogen inhibition could explain poorer growth of *C. scindens* in the anaerobic chamber, but that riboflavin supplementation continues to enhance *C. scindens* growth even when this inhibition is relieved.

### *C. scindens* breaks down riboflavin into lumichrome anaerobically

**Fig 4:**
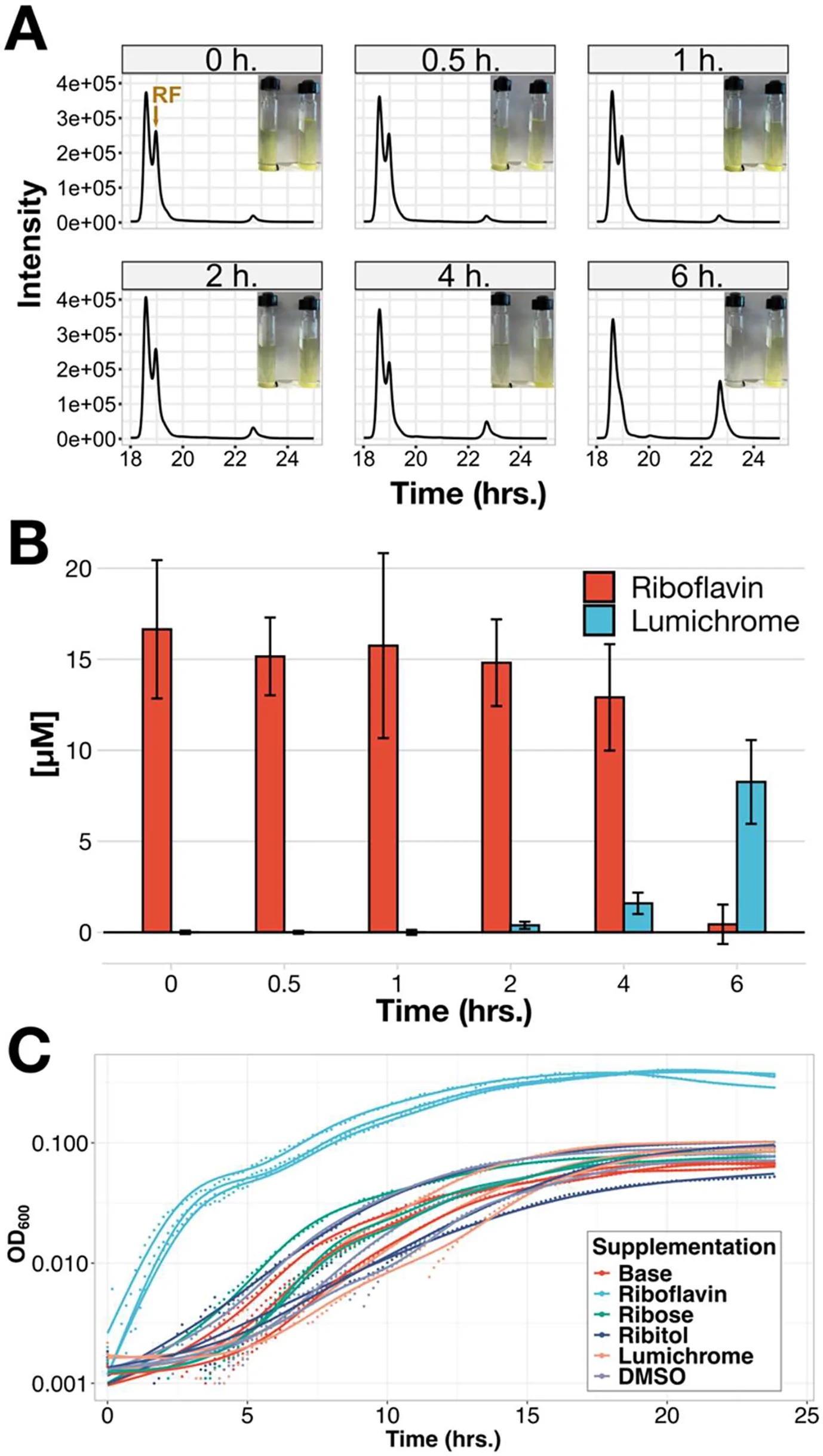
*C. scindens* catabolizes riboflavin to lumichrome, but neither lumichrome nor five-carbon side-chain catabolites enhance growth. (**A**) HPLC traces showing the riboflavin peak (second peak at ~ 19 m labeled “RF”) and an unidentified peak (~23 m). Traces are representative of *n* = 3 biological replicates. Representative images of an inoculated culture (left) and an uninoculated control (right) are shown in insets. Headers show time post-inoculation (from 0 to 6 h). (**B**) Quantitation of riboflavin and lumichrome in *C. scindens* culture supernatants over time by targeted mass spectrometry. Error bars are 2 × SE of *n* = 3 biological replicates. (**C**) Growth curves showing *C. scindens* growth on base medium (0.13 µM riboflavin) or medium supplemented with 17.5 µM riboflavin (teal), 200 µM ribose (green), 200 µM ribitol (dark blue), 200 µM lumichrome (salmon), or a DMSO control (light blue). Each trace is one biological replicate (*n* = 3); lines are non-parametric smooth fits (generalized additive model).

*C. scindens* could also be degrading flavins during growth, requiring replenishment beyond what is necessary to account for dilution. In support of this hypothesis, we noticed that while the 17.5 µM riboflavin medium was bright yellow, *C. scindens* cultures turned the medium clear over the course of 6 h. Uninoculated controls remained brightly colored over the same time period (Fig. 4A, insets).

We therefore performed HPLC on supernatants from Hungate tube cultures sampled at between 0 and 6 h of growth (see Materials and Methods). We identified a peak at 19 min as riboflavin, which decreased over this time period; an unknown peak 3 to 4 min later had the opposite trend, increasing exponentially in height over the course of 6 h (Fig. 4A). The unknown peak was also present at a low level at time zero. Commercially available riboflavin typically contains low levels of breakdown products such as lumichrome, which forms upon exposure to light and oxygen.

Using liquid chromatography-mass spectrometry with fragmentation (LC-MS/MS), we therefore tested whether lumichrome and/or alloxazine were formed during growth. Riboflavin, lumichrome, and alloxazine were readily distinguishable by mass, fragmentation, and elution time (Fig. S1). Alloxazine was not detected in pre- or post-inoculation media. In both our HPLC and LC-MS/MS analyses, lumichrome was detected in media at the same retention time as our standard (Fig. S2A). We additionally confirmed the identity of the unknown peak in our HPLC data by collecting fractions and measuring lumichrome via LC-MS/MS (Fig. S2B). When quantitated over the course of *C. scindens* growth, lumichrome showed the expected trend based on the HPLC data (Fig. 4B), indicating that lumichrome was indeed the major product. Approximately half of the provided riboflavin (in moles) appeared to be catabolized to lumichrome over the first 6 h of growth.

The only characterized bacterial pathway for degrading riboflavin to lumichrome is found in *Microbacterium maritypicum* (34) and *Devosia riboflavina* (35) (formerly *Pseudomonas riboflavina*). However, it requires oxygen for riboflavin cleavage by the monooxygenase RcaE, and the pathway therefore does not proceed anaerobically even when reconstituted *in vitro*. As *C. scindens* is a strict anaerobe, it is unsurprising that a BLASTP search failed to find any significant similarity to RcaE in its genome. This is consistent with the reported taxonomic distribution of the aerobic riboflavin degradation pathway in the Actinomycetota and the alphaproteobacterial genus *Devosia* (35). There have been reports of gut microbes that degrade riboflavin to lumichrome (56), but these strains were never identified and appear to have been lost. Interestingly, one anaerobic isolate from goat rumen, *Faecalicatena fissicatena*, which is also a member of the *Lachnospiraceae*, has been reported to degrade riboflavin, but to a different major product, hydroxyethylflavin (HEF, or “ethanol lumiflavine”) (57). This is therefore the first report of a taxonomically identified microbe capable of anaerobically degrading riboflavin to lumichrome.

### Neither lumichrome nor five-carbon catabolites of riboflavin enhance *C. scindens* growth

Riboflavin has a ribityl chain attached to its isoalloxazine ring system, which is missing in lumichrome. *M. maritypicum* and *D. riboflavina* cleave this chain using an aerobic, oxidative process that releases ribose, which can serve as a carbon and/or energy source (34, 35). In our experiments, any ribose or other carbon source liberated would be orders of magnitude lower in concentration than the fructose in our medium (17.5 µM vs 25 mM); however, it might contribute disproportionately to growth if riboneogenesis from fructose were costly or inefficient in *C. scindens*. Alternatively, lumichrome, rather than riboflavin itself, might actually enhance *C. scindens* growth. This possibility is especially important to test as lumichrome is a trace contaminant of commercially available riboflavin.

To assess these possibilities, we grew *C. scindens* in DM with an excess (200 µM) of ribose, ribitol (the expected product of non-oxidative cleavage), or lumichrome. Lumichrome is much more hydrophobic than riboflavin, limiting its solubility, so we prepared its stock solution in DMSO and compared it to a vehicle control. We saw no growth enhancement in any condition other than 17.5 µM riboflavin supplementation (Fig. 4C), even though the *C. scindens* genome does encode predicted homologs of the *rbsABC* ribose import system.

This result indicates that riboflavin itself enhances *C. scindens* growth, not lumichrome, and does so by a mechanism other than providing ribose or ribitol. Importantly, it does not exclude a function for all possible catabolites of the ribityl chain. However, it does show that the anaerobic degradation process differs in function and/or mechanism from the previously described aerobic pathway.

### A specific gene neighborhood is induced during *C. scindens* growth on high riboflavin

**Fig 5:**
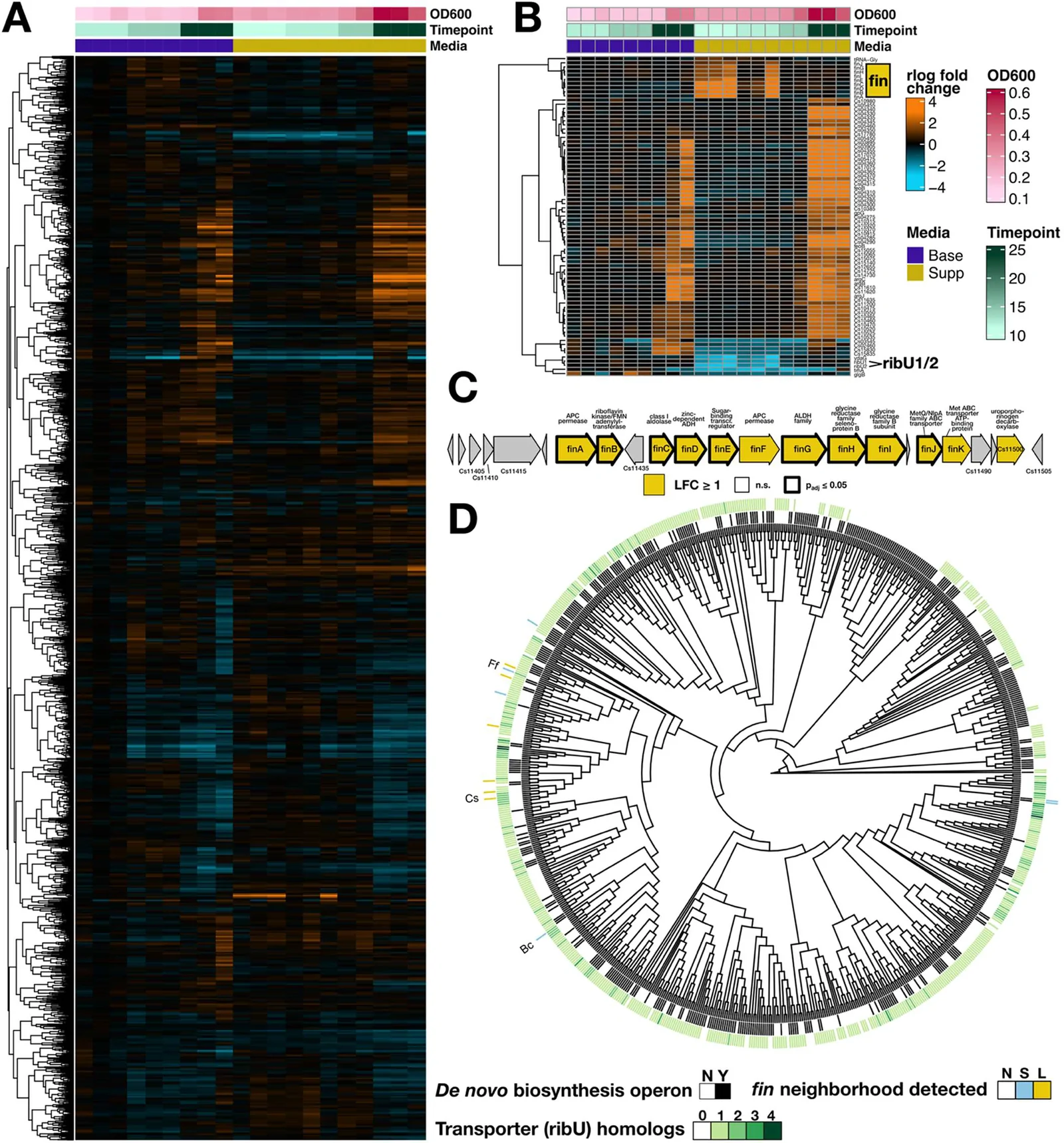
A single gene neighborhood, with sporadic distribution across *Lachnospiraceae*, is induced on high riboflavin. (**A**) Clustered heatmap showing all transcripts under base media (0.13 µM riboflavin, blue) and supplemented conditions (17.5 µM, orange). Counts were rlog-normalized using DESeq2. (**B**) Transcripts with significant (adjusted *P* ≤ 0.05, log_2_ fold change > 2) effects by medium. The *fin* neighborhood genes are highlighted with an orange label. The riboflavin transporters *ribU1* and *ribU2* are also labeled. (**C**) *C. scindens* genomic region containing most induced genes. Genes are highlighted in orange if they had a log_2_ fold change of at least 1, and outlined in black if they were found to be significant (by the criteria in panel **B**). (**D**) Tree of *Lachnospiraceae* reference genomes showing, from inside to outside, distribution of the *de novo* riboflavin biosynthesis operon (black), number of *ribU* homologs (olive), and whether the long (orange, L) or short (teal, S) version of the *fin* neighborhood was detected. Taxon labels: Cs, *Clostridium scindens*; Ff, *Faecalicatena fissicatena*; Bc, *Blautia coccoides*.

Since the above experiments pointed to a presently unknown pathway for riboflavin degradation, we performed transcriptomics on *C. scindens* cultures during log-phase growth and stationary phase, in either supplemented (17.5 µM) or base (0.13 µM) riboflavin. Most transcripts had similar trends that were driven mainly by the phase of growth (Fig. 5A). However, a small subset of transcripts did show strong, statistically significant differential expression across media (Fig. 5B, *p*_adj_ ≤ 0.05 and log_2_(fold change)>2, DESeq2 Wald test).

Both predicted riboflavin transporters (*ribU1* and *ribU2*) were upregulated in the base medium compared to the supplemented medium, providing additional evidence that these indeed function in riboflavin import. Additionally, one predicted homolog of the *Bacillus* stress-response gene *ydaJ* was highly correlated with *ribU1/2* expression. While the function of YdaJ is unknown, we noted that it is a predicted flavin-dependent pyridoxamine-5-phosphate (PNP) oxidase that converts PNP to the active pyridoxal-5-phosphate (PLP) and would therefore bind both FAD and pyridoxamine.

The genes upregulated under riboflavin supplementation, in contrast, were almost all located in a separate, single neighborhood in the *C. scindens* genome (Fig. 5C). Coverage plots indicated that these likely formed at least three distinct transcriptional units (Fig. S3). As most of these genes had no annotated name, we have labeled them here as *finA-K,* for “flavin induced neighborhood.” This neighborhood encoded a predicted homolog of the bifunctional riboflavin kinase/FMN adenylyltransferase RibF (*finB*), as well as a predicted Class I aldolase (*finC*), an alcohol dehydrogenase (*finD*), an aldehyde dehydrogenase (*finG*), and two genes homologous to glycine/betaine/sarcosine reductase subunits (*finHI*).

Because a *F. fissicatena* isolate was previously reported to catabolize riboflavin to the related product HEF, we also used MMseqs2 to identify reciprocal best hits between *F. fissicatena* and *C. scindens* coding sequences. This revealed that this strain of *F. fissicatena* indeed encodes a similar but shorter neighborhood, lacking homologs of the aldehyde dehydrogenase and the glycine/betaine/sarcosine reductase (*finGHI*).

### Orthologous neighborhoods can be detected across related *Lachnospiraceae*

We next performed a genomic analysis to identify other *Lachnospiraceae* with the same gene neighborhood and to compare its distribution to riboflavin auxotrophy and transport (Fig. 5D). Using the OrthoFinder pipeline (58), we built a tree of 792 high-quality *Lachnospiraceae* isolate and metagenomically assembled genomes (Table S4) from the Genome Taxonomy Database (GTDB), including the genomes of the five strains we tested in our initial experiment plus one outgroup (*Vallitalea_A okinawensis*); we also assembled orthogroups and constructed orthogroup gene trees.

We found that the *de novo* riboflavin biosynthetic pathway had a patchy, heterogeneous distribution among the *Lachnospiraceae*, with 49% of the *Lachnospiraceae* predicted to be auxotrophs based on lacking this pathway (Fig. 5D, black lines). Not all *Lachnospiraceae* encoded *ribU*, but, matching a previous observation (16), the *ribU* transporter gene was almost always present when the *de novo* pathway was missing. Furthermore, as we saw in *C. scindens* and *B. coccoides*, many other *Lachnospiraceae* had a second *ribU* paralog (12.5%), with some diverged *Lachnospiraceae* having even a third or fourth (Fig. 5D, green colors).

Several of the genes in the *fin* neighborhood, such as the alcohol dehydrogenases, were assigned to very large orthogroups that contained multiple paralogs per genome, making it difficult to assess functional conservation. We therefore used gene trees to refine these larger families, using the *C. scindens* and *F. fissicatena* sequences as guides. We focused on *finBCDE* as these were present at a single copy in both genomes; *F. fissicatena* has only a single ortholog of *finA/F,* and, as noted above, lacks all other *fin* genes.

Notably, the *C. scindens* and *F. fissicatena finBCDE* sequences belonged to smaller monophyletic clades containing only 10–20 members each. In two cases, *finB* and *finC*, these were separated from other sequences in the orthogroup by a long branch (Fig. S4), consistent with functional divergence. We called orthologs for *finBCDE* based on these subtrees and provisionally classified genomes with all four orthologs as *fin+* (Table S5).

This analysis yielded 10 additional representative genomes besides *C. scindens* and *F. fissicatena* that encoded *fin* orthologs, including our isolate of *B. coccoides* (Fig. 5D, orange and teal lines). The *fin* orthologs had a sporadic distribution across *Lachnospiraceae*, possibly indicating horizontal transfer, but were most concentrated in a subclade that includes the genera *Dorea*, *Mediterraneibacter*, and *Muricomes*. We found cases of both the longer *C. scindens-like* neighborhood with *finGHI* homologs (orange lines; e.g., *Muricomes intestini*), as well as the shorter *F. fissicatena-*like neighborhood variant without these (teal lines; e.g., *Mediterraneibacter hominis*). The *F. fissicatena* and *M. hominis* neighborhoods were both flanked by another aldehyde-alcohol dehydrogenase gene that was not in the same orthogroup as any of the original *fin* genes. Thus, at least two separate variants of this neighborhood appear to be carried by multiple *Lachnospiraceae* (Fig. S5).

### Genomic and metabolomic data point to gut gram-negative bacteria as a potential riboflavin source for *Lachnospiraceae in vivo*

If *C. scindens*, and potentially other *Lachnospiraceae*, are so dependent on a vitamin that they also degrade, that vitamin should be abundant in its local context. However, in mammals, dietary riboflavin is known to be efficiently absorbed in the upper small intestine. Some have argued that unabsorbed riboflavin could persist in the large intestine (59), but according to recent survey data (60), the average American over 20 years old consumes only 1.92 ± 0.034 mg of riboflavin daily, well below the estimated maximum of 27 mg that could be absorbed in a single dose (61). This suggests that either *C. scindens* is metabolizing riboflavin that crosses from the blood to the colon (as is observed for other bloodborne nutrients such as lactate and urea) (10), or there is an alternative source of riboflavin in the gut, possibly microbial in origin.

**Fig 6:**
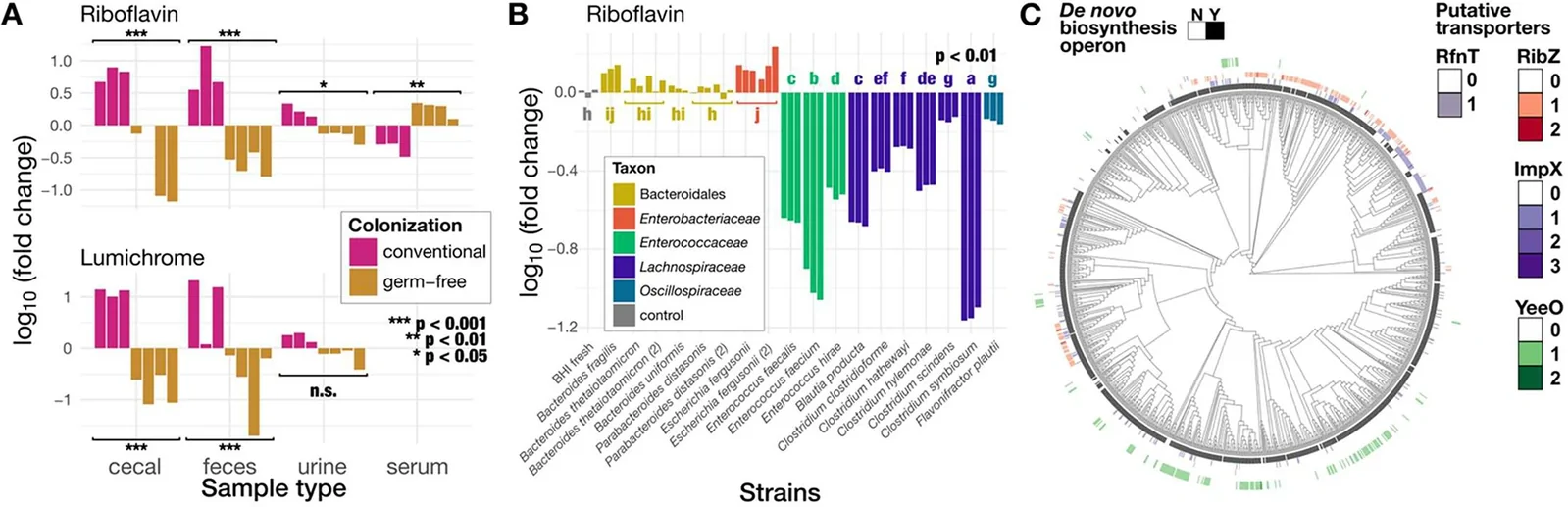
Metabolomic and genomic data support a role for riboflavin cross-feeding in the gut. (**A**) Metabolomics data from Han et al. (62) showing *in vivo* measurements of riboflavin (top) and lumichrome (bottom) across four different tissues in colonized (navy) and germ-free (orange) mice. Measurements were normalized to the average of each group to show log-fold changes. Significance was assessed using a linear model with a log link. (**B**) Metabolomics data from Ho et al. (63) showing riboflavin concentrations in spent medium (BHI) for gut isolates from several different families (colors). Uninoculated BHI is shown in gray and was used to normalize the other measurements. A compact letter display shows significant differences of marginal means between groups; groups that do not share letters are significantly different at *P* < 0.01. Significance was assessed using a linear model with a log link. (**C**) Tree of *Bacteroidales* genomes with the riboflavin *de novo* pathway (black) and copies of putative transporters (RfnT homologs, teal; RibZ, red; ImpX: blue; YeeO, green) highlighted in rings; note that YeeO is a potential exporter.

We re-analyzed metabolomic data from a study (62) that compared germ-free to conventionally colonized mice and found that riboflavin was significantly more abundant in the intestines, ceca, and feces of colonized mice than in those of germ-free mice (Fig. 6A). This is consistent with gut microbes overproducing riboflavin *in vivo*. Han et al. (62) also sampled serum, where a modest but significant trend in the opposite direction was observed (Fig. 6A), suggesting that microbial riboflavin is not substantially absorbed by the host. Finally, Han et al. also measured a metabolite that they identified as lumichrome, which was also significantly higher in colonized mice (Fig. 6A). Since riboflavin can be degraded to lumichrome in the presence of UV light and oxygen, we cannot exclude the possibility that this mainly reflects increased riboflavin abundance; however, this is consistent with previous results where HEF was found only in colonized and not germ-free rabbits (38).

To determine possible microbial sources of riboflavin, we turned to metabolomic data from a recent set of *in vitro* culture experiments that sampled several diverse gut isolates grown in rich medium (BHI), including members of the genera *Escherichia* and *Bacteroides* (63). Riboflavin in spent medium from *B. fragilis* and *E. fergusonii* was indeed significantly higher after culturing than in uninoculated controls (Fig. 6B). Further supporting the idea that many *Lachnospiraceae* are riboflavin auxotrophs and/or have a high requirement for this vitamin, all *Lachnospiraceae* tested in this study depleted significant quantities of riboflavin from BHI, while none of the tested gram-negative bacteria did so.

We finally compared these patterns to those obtained from an analysis of 1,291 genomes (Table S6) from the most abundant gram-negative clade, the *Bacteroidales,* plus three outgroups (*Flavobacterium psychrophilum, Flavobacterium columnare*, and *Flavobacterium johnsoniae*). In contrast to the *Lachnospiraceae*, we found that *de novo* biosynthesis was highly conserved in this clade, and riboflavin import was much less so. However, some predicted transporters had a sporadic distribution, suggesting transfer into the clade (Fig. 6C). Interestingly, *Bacteroidaceae* specifically were more likely to have homologs of the *E. coli* MATE transporter YeeO, which has been shown in *E. coli* to be a flavin exporter (64). Further work will be required to confirm the function of this homolog in *Bacteroidaceae*.

Overall, these observations are consistent with a model in which *Bacteroidaceae* and *Enterobacteriaceae* overproduce riboflavin in the gut, which is then taken up by gram-positive bacteria, and additionally degraded to either HEF or lumichrome by specific gut *Lachnospiraceae*.

## DISCUSSION

### Anaerobic catabolism of riboflavin by *Lachnospiraceae*

We found that *C. scindens* growth was enhanced by riboflavin supplementation, that this enhancement continued up to at least 90 µM, and that *C. scindens* also degraded approximately half of the riboflavin in the medium to lumichrome over the course of 6 h of log-phase growth. Furthermore, a single gene neighborhood was strongly induced under high (17.5 µM) riboflavin, relative to a 0.13 µM medium control. While it has previously been suggested that lumichrome is a gut bacterial metabolite and not a host-derived molecule (65), this is the first report to identify a specific microbe, *C. scindens*, that is capable of degrading riboflavin to lumichrome without the use of oxygen. The only currently known riboflavin degradation pathways both require oxygen for key catalytic steps. In *M. maritypicum* and *D. riboflavina*, riboflavin is split to ribose and lumichrome using the flavin monooxygenase RcaE (34, 35), while in *S. meliloti*, the flavin destructase BluB uses oxygen to split the isoalloxazine ring itself, yielding an intermediate in B_12_ biosynthesis (66, 67). This points to a new and different mechanism for flavin side-chain cleavage by *C. scindens*.

The only other anaerobe capable of riboflavin degradation that has been identified to date is also a member of the *Lachnospiraceae*. In 1970, an isolate of *Faecalicatena fissicatena* was collected that produced the alternative product hydroxyethylflavin (HEF, or also “ethanol lumiflavine”) (57). However, no candidate pathways were ever identified. Using comparative genomics, we found that this specific isolate of *F. fissicatena* is one of the few *Lachnospiraceae* to have a variant of the *fin* neighborhood. Furthermore, consistent with a different product being performed, three genes in the *C. scindens* pathway are missing from *F. fissicatena*, including two genes in the glycine/betaine/sarcosine reductase family. Intriguingly, enzymes in this family break C–N bonds, and HEF differs from lumichrome by having a two-carbon chain bound to a nitrogen on the isoalloxazine ring. This comparison therefore suggests a mechanism in which a triose is liberated first in both organisms by *finBCD*, followed by cleavage of a two-carbon unit in *C. scindens* by *finHI* that is retained in *F. fissicatena*. While further work will be required to fully characterize each gene in the *fin* neighborhood, the existence of these variants could also explain why previous studies found multiple riboflavin catabolites, such as HEF and formylmethylflavin (FMF), in human urine (36, 37) and the ceca of colonized rabbits (38).

We did also observe homologs of the *fin* genes in *B. coccoides*, and like *C. scindens*, it appears to be a riboflavin auxotroph based on its genome content. However, riboflavin supplementation did not restore its growth. Future work is required to determine the growth factors it may be missing on our defined medium, or, alternatively, inhibitory factors that may be present.

### Why does *C. scindens*, a riboflavin auxotroph, degrade riboflavin?

Ribose, ribitol, and lumichrome failed to enhance *C. scindens* growth. Therefore, unlike what was previously observed for the aerobic riboflavin degradation pathway, five-carbon substrate utilization does not appear to explain the large growth benefit we observed. This likely reflects that different catabolites are formed by the anaerobic pathway; the comparison to *F. fissicatena*, in particular, strongly suggests that the side chain is not liberated in a single step. Further investigation will focus on which specific products are generated and how these might contribute to growth.

Another explanation, which is not mutually exclusive, could be that riboflavin degradation contributes to redox balance. During carbon oxidation, FAD and FMN are reduced to FADH_2_ and FMNH_2_; in anaerobes, these are typically re-oxidized via pathways that include donating electrons to hydrogenase (carried by flavodoxins), or to substrates such as fumarate (68), urocanate (69), or, in the case of *C. scindens*, bile acids (47). However, in the absence of a suitable electron acceptor, oxidized flavins may be unavailable for key central carbon metabolic enzymes such as, for example, pyruvate-flavodoxin/ferredoxin oxidoreductase (PFOR). An excess of reactive reducing equivalents, or “reductive stress,” could also have other deleterious effects (70). Cells might therefore benefit from catabolizing excess reduced flavins into a product such as HEF or lumichrome, especially when oxidized riboflavin is abundant. The concentration gradient would likely favor non-energetic export of riboflavin catabolites; we note that *finA/F* are predicted to be permeases. This could be understood as a type of directed overflow metabolism (71). Indeed, flavin biosynthetic intermediates are already known to be targeted for overflow metabolism because of their reactivity (32, 72).

If this were true, we would expect the pathway to be selective for reduced flavins such as FADH_2_ or FMNH_2_. In support of this hypothesis, we note that the *fin* neighborhood contains an enzyme annotated as a bifunctional FAD synthetase, *finB*. While this family of enzymes is named for its ability to convert riboflavin to FMN and then to FAD, prokaryotic bifunctional FAD synthetases can also have FAD pyrophosphorylase activity, meaning that they generate FMN from FAD. Furthermore, one such bifunctional FAD synthetase, purified from *Streptococcus pneumoniae*, was indeed shown to be highly selective for de-adenylylating the reduced form, FADH_2_ (73), and a similar preference was observed in a FAD synthetase from *B. subtilis* (74). Notably, this reverse reaction can potentially generate ATP from pyrophosphate. An interesting future direction would therefore be to test whether the FinB protein indeed functions as an FADH_2_-specific pyrophosphorylase.

Another observation supporting a role in redox has to do with the lumichrome produced by *C. scindens*. Oxidized lumichrome, such as riboflavin, is bright yellow, and because of its lower solubility, aerobic catabolism of riboflavin to lumichrome typically yields a bright yellow precipitate, as observed for *M. maritypicum* (34). We did not observe this in our *C. scindens* cultures. Fully reduced lumichrome, in contrast, absorbs mostly in the UV spectrum (75) and therefore has little visible color, meaning that the lumichrome being formed in our culture supernatants could be in the reduced form.

Finally, anaerobic riboflavin degradation to lumichrome could simply be due to metabolic damage (76), with the *fin* neighborhood playing a separate role in flavin metabolism. While this would not explain the conservation of *fin* genes in *F. fissicatena*, nor why the major products are different, it is also true that cofactors can be damaged by either non-enzymatic spontaneous or enzymatic side reactions (32). Unlike, for example, nicotinate cofactors, flavins can exist in either singly or doubly reduced forms, making reactions that depend on radical chemistry more likely. Certain flavin biosynthesis intermediates are indeed known to be labile (32, 72), and in the most famous example of non-enzymatic degradation, riboflavin can decompose in the presence of light and oxygen. Photolysis cannot explain our current results (note that we did not observe bleaching in uninoculated controls), but does speak to the general reactivity of flavins.

On the other hand, despite this potential for reactivity, pulse-chase labeling experiments in prototrophic *E. coli* and *B. subtilis* strains have found low baseline rates of flavin damage. In these experiments, the “pool turnover” rate of cofactors was measured, and FAD and FMN were actually found to be some of the most stable (compared to, e.g., folates or ATP), with most of the turnover accounted for by dilution caused by cell division (77). What difference in the cellular environment and/or growth conditions of *C. scindens*, then, would favor much higher rates of flavin damage, with nearly half the riboflavin converted into lumichrome? One possibility is that the above pulse-chase experiments were performed aerobically and therefore also in the presence of a good electron acceptor. It remains to be determined whether flavin pool turnover in a prototroph would remain so low under anaerobic and/or reductive stress conditions, which might accelerate metabolic damage.

### Vitamin catabolism could be an underappreciated contributor to the gut microbial food web

Our reanalysis of previous metabolomic data shows that riboflavin is likely produced by gut microbes and potentially cross-fed from gram-negative to gram-positive bacteria. Most previous studies of vitamin metabolism in gut microbes have focused on auxotrophy as a main potential driver of cross-feeding interactions. Vitamin catabolism, in contrast, is largely understudied; as mentioned above, the aerobic pathway for riboflavin degradation was only elucidated more than 70 years after it was first described in soil isolates. Beyond riboflavin, catabolism may also be relevant for other vitamins that are produced and consumed in the gut. Pyridoxal, nicotinate, and especially biotin are elevated in colonized vs germ-free mouse guts (78), and there is *in vitro* evidence for gut microbial production and uptake of thiamine (18, 79) and folate (18).

While most of our supplementation experiments were performed with 17.5 µM riboflavin, the concentration *in vivo* may not need to be as high if flavins are continuously secreted by other microbes. Microbes such as *E. coli*, *P. fluorescens*, and *B. subtilis* have all been observed to overproduce and excrete flavins into the supernatant (49). Flavin production is not tightly regulated at the level of feedback inhibition (49), perhaps because flavin biosynthetic intermediates can be toxic to the cell (72). *Lachnospiraceae* might also elicit riboflavin overproduction in other microbes, either actively (e.g., via signaling) or simply by lowering its extracellular concentration.

### Riboflavin degradation products may have further impacts on both microbiome and host

There is precedent for microbial sensing of lumichrome; for example, the LasR quorum sensing receptor from *P. aeruginosa*, whose preferred ligand is an acyl-homoserine lactone, can also be activated by both riboflavin and, especially, lumichrome, although this activation is weaker than for the preferred ligand (80). *P. aeruginosa* is also found in soils; production of lumichrome stimulates plant root growth (81) and is thus an important function of rhizosphere bacteria (potentially including *Devosia*). A pathway for the complete degradation of lumichrome to organic acids and ammonia was also just identified in *Nocardioides simplex*, raising the possibility that lumichrome may be further metabolized in the gut, though the *N. simplex* pathway is also dependent on oxygen (82).

Perhaps even more importantly, in addition to its role as a vitamin and cofactor component, riboflavin and related metabolites are known to be sensed by the mammalian immune system. Mucosal-associated invariant T cells, which are enriched in the mammalian gut, express a specific narrow range of T-cell receptors (TCRs) that bind the MHC class I-like antigen-presenting protein MR1. MR1 binds small molecules related to B vitamins (83) and, when bound to adducts of riboflavin biosynthetic intermediates, activates MAIT cells (84). Since humans do not synthesize riboflavin *de novo*, these intermediates are necessarily microbial in origin and are associated with fast-growing pathogens. In contrast, MR1 bound to riboflavin itself inhibits MAIT cell activation; as colonized guts are much higher in riboflavin, this may help to control MAIT cell activation.

How do flavin catabolites fit into this picture? Interestingly, a recent study showed that lumichrome and other breakdown products, such as FMF and lumiflavin, actually bind MR1 with higher affinity than riboflavin. However, unlike the riboflavin biosynthetic intermediates, these catabolites competitively inhibit MAIT cell activation (85). The study authors explained this phenomenon by referring to lumichrome as a “host metabolite,” pointing out that it is found in human blood. However, studies on rat tissue have argued against an animal origin of lumichrome (65). As we have demonstrated, human gut microbes can catabolize riboflavin to lumichrome. Others have implicated gut microbes in the degradation of riboflavin to products such as HEF (38) and FMF (36), noting that (as we found in our re-analysis of metabolomic data) riboflavin catabolites are enriched specifically in colonized vs germ-free animals. An alternative interpretation of the above findings is that commensal bacteria that catabolize riboflavin therefore inhibit MAIT cell activation. This could be another mechanism by which they contribute to an anti-inflammatory tone in the gut beyond, for example, the production of short-chain fatty acids (SCFAs). Future work, potentially in a gnotobiotic system, will be required to directly test the *in vivo* impact of microbial riboflavin degradation on the host.

## MATERIALS AND METHODS

### Bacterial strains

Initial growth curve experiments were performed with five representative members of the *Lachnospiraceae* family: *Robinsoniella peoriensis* B-23985*, Anaerostipes caccae* DSM 14662*, Blautia coccoides* ATCC 27340*, Dorea formicigenerans* ATCC 27755*,* and *Clostridium scindens* ATCC 35704. Strain designations, culture collection sources, and accession numbers are provided in Table S1. All strains were verified by amplifying the 16S rRNA gene using bacterial universal primers 27F and 1492R, followed by Sanger sequencing, and maintained as glycerol stocks (16.6% vol/vol) at −80°C.

### Pre-culture conditions

Strains were struck onto agar plates containing brain heart infusion supplemented with hemin, menadione, cysteine, and resazurin (BHI-CHK) (86) and incubated anaerobically inside a Coy anaerobic chamber maintained with an atmosphere of 85% nitrogen, 10% carbon dioxide, and 5% hydrogen. Because hydrogen is consumed by the catalyst, the working hydrogen concentration was typically 2–3%. Three independent colonies per strain were inoculated into BHI-CHK broth and grown anaerobically for 24 h prior to transfer into a chemically defined medium.

### Chemically defined medium formulation

A chemically defined medium (DM) was adapted from Devendran et al. (41) with some modifications. We chose fructose as the carbon source because preliminary analysis of RefSeq and KEGG annotations showed that fructokinase and fructose-specific PTS transporters appeared to be common among *Lachnospiraceae*, and because fructose is a common component of dietary fibers such as inulin. We also used 100× vitamin and mineral stock solutions, which were based on Wolin and Wolfe’s formulation (87). For minerals, we used a commercial supplement (ATCC, MD-TMS) that used EDTA as a chelator instead of nitrilotriacetic acid. We prepared our own vitamin stock solutions; differing from Wolin and Wolfe’s original formulation, we provided vitamin B_6_ in three forms—pyridoxine, pyridoxal, and pyridoxamine—as some *Lachnospiraceae* lacked the predicted enzymes to activate one or more of these to pyridoxal-5-phosphate (PLP). Finally, our medium did not include resazurin.

The final medium therefore consisted of the following (final concentrations): fructose (25.00 mM); NaHCO_3_ (45.00 mM); NaPO_4_ (36.60 mM); KPO_4_ (22.00 mM); NaCl (8.556 mM), NH_4_Cl (18.7 mM); biotin (0.00008 mM); folic acid (0.00005 mM); pyridoxine (0.00049 mM); thiamine (0.00015 mM); riboflavin (0.00013 mM); nicotinic acid (0.0004 mM); pantothenic acid (0.00021 mM); cobalamin (7.40e^−7^ mM); p-aminobenzoic acid (0.0036 mM); (DL)-alpha lipoic acid (0.00024 mM); pyridoxal (0.001 mM); EDTA (6.8 µM); MgSO_4_ (48 µM); MnSO_4_ (12 µM); FeSO_4_ (1.44 µM); Co(NO_3_)_2_ (1.36 µM); CaCl_2_ (3.6 µM); ZnSO_4_ (3 µM); CuSO_4_ (0.16 µM); AlK(SO_4_)_2_ (0.156 µM); H_2_BO_3_ (0.64 µM); Na_2_MoO_4_ (0.164 µM); Na_2_SeO_3_ (0.0232 µM); NaWO_4_ (0.12 µM); NiCl_2_ (0.336 µM); all 20 amino acids (0.2 mM); Na_2_S (1.2 mM); and L-cysteine (1.5 mM). We also prepared a version of this defined medium supplemented with acetate (DM + Ace) by adding sodium acetate at 45 mM.

For experiments conducted inside the anaerobic chamber, all media were prepared aerobically, filter sterilized, and pre-reduced inside the anaerobic chamber at least overnight prior to inoculation.

### Chemically defined media growth curves

Growth assays were conducted in sterile 96-well plates with a final working volume of 200 µL per well. Following pre-culture growth, 2 mL of each BHI-CHK culture was harvested by centrifugation at 4,000 rpm for 10 min inside the anaerobic chamber. Supernatants were decanted, and cell pellets were resuspended in 10 mL of pre-reduced DM. Cultures were inoculated by transferring 20 µL of the resuspended cells into 180 µL of fresh DM. Plates were sealed with a gas-permeable Breathe-Easy film (Diversified Biotech BEM-1) and transferred to an automated plate stacker connected to an Agilent microplate reader. Optical density at 600 nm (OD_600_) was measured every 10 min for 24 h. Prior to each measurement, plates were shaken for 30 s using a double-orbital motion to homogenize the culture. As it was difficult to perform subcultures from a plate sealed with a Breathe-Easy film without aerosol contamination, a parallel replicate plate was sealed with a Breathe-Easier film and incubated adjacent to the plate stacker under the same conditions.

Following 24 h of incubation, 20 µL from the replicate plate was transferred into 180 µL of fresh pre-reduced DM into two new 96-well plates. This subculturing procedure was repeated for a total of three sequential transfers under identical anaerobic and measurement conditions. Wells were blanked based on the median value of all blank wells per media type and plate. Growth curve parameters (e.g., carrying capacity and growth rate) were fit to a logistic equation using Growthcurver (88):


$$OD600=k\left(\frac{1}{1+\left(\frac{k-N_{0}}{N_{0}}\right)e^{-rt}}\right)$$

Here, carrying capacity (*k*) corresponds roughly to the “maximum” OD_600_, *N_0_* to the initial population size, and *r* to the growth rate.

For visualization, growth curves were fit to a generalized additive model (GAM) using the mgcv package (89) in R (90). A separate GAM was fit using restricted maximum likelihood (REML) for each combination of species, subculture, and media, using a Gaussian model with a log-link. OD_600_ was modeled as a smooth function of time (five knots), with biological replicate modeled as a random effect. Standard errors (SE) of the mean were calculated, correcting for uncertainty in smoothing parameters; the 95% CI was obtained by taking 1.96 × SE. Carrying capacities (*k*) were calculated per technical and biological replicate for each combination of species, subculture, and media, then summarized using a weighted GAM, again modeling biological replicate as a random effect. Weights were the reciprocal of the squared standard error in *k* reported by GrowthCurver, which allowed us to incorporate uncertainty in model fitting. Finally, estimated marginal means and their 95% confidence intervals were computed directly using the emmeans package (91).

### Comparative genomics in cultivated strains

Genomes from *D. formicigenerans*, *C. scindens*, *B. coccoides,* and *A. caccae* were obtained from the NCBI Refseq database. In addition, the genome from *R. peoriensis* was sequenced and assembled as part of this study, using a combination of short-read Illumina and long-read Oxford Nanopore sequencing.

Assembly was performed according to the guidelines of Wick, Judd, and Holt (92). Raw short reads for *R. peoriensis* were processed using fastp (93) v0.24. The raw long reads were subsampled into independent read sets with Trycycler (94) v0.5.5, then assembled with Flye (95) v2.9.3, Minipolish v0.1.3 (96), and Raven (97) v1.8.3. Each assembly was clustered and reconciled with Trycycler, and contigs were manually reviewed. Six contigs were excluded due to large gaps and poor alignments, indicating large structural errors. All contigs that passed a manual check were then processed with multiple sequence alignment, and a consensus was generated with Trycycler. The final consensus was polished with Medaka (98) v2.0.0 using long reads and further refined with short reads using Polypolish (99) v0.5.0. Finally, one additional round of polishing was completed with Polca (100) v0.2.1.

All genomes were annotated using the anvi’o platform v8 (101). KEGG Orthologs (KOs) were assigned using the anvi-run-kegg-kofam workflow, which identifies KOs with a hidden Markov model approach using adaptive cutoff adjustment to improve sensitivity (KEGG release 107.0, September 2023) (45). The default parameters were used unless otherwise specified.

A binary presence–absence matrix of KOs was generated from anvi’o annotations for all genomes, with KOs scored as present if at least one copy was detected in a given genome. The resulting matrix was used for comparative genomic analyses across strains.

### Riboflavin supplementation

To assess the effect of the riboflavin availability on growth, the chemically defined medium was supplemented with riboflavin at 17.5 µM. We used a saturated stock solution of riboflavin in water, heating to 60°C for 4–6 min before filter sterilizing. Using absorbance to measure the concentration, we found that 175 µM riboflavin remained in solution after sterilization, consistent with the reported solubility limit in water. This stock solution was made fresh on the day of each experiment in order to avoid possible degradation. We used this stock at 10× to yield media with 17.5 µM riboflavin. Growth curve experiments were performed inside the Coy anaerobic chamber; growth curve experiments, incubation, and data acquisition were performed using the same protocol as described in previous sections.

### Riboflavin titration

Three biological replicates of *C. scindens* were initially cultured in CDM supplemented with 200 µM of riboflavin inside the Coy anaerobic chamber. After 48 h of inoculation, cultures were diluted with riboflavin-free CDM to a final OD_600_ of 0.5. Subsequently, 20 µL of the resuspended cultures were inoculated into individual wells of a 96-well plate containing 180 µL of CDM with various concentrations of riboflavin (200, 150, 120, 90, 60, 30, 15, and 1.5 µM).

Because of the higher concentrations in this experiment, riboflavin-supplemented media were prepared from a 10 mM stock solution dissolved in DMSO. To ensure that all treatment conditions contained the same final concentration of DMSO, individual riboflavin working stock solutions were prepared by diluting the 10 mM stock solution in DMSO. Each working stock was then added to CDM in the appropriate volume to achieve the desired final riboflavin concentration. The 10 mM riboflavin stock solution was previously purged with gas mix before being added to CDM in glass beakers to prepare the different riboflavin concentrations inside the anaerobic chamber. The prepared medium was then filter sterilized prior to inoculation. Following inoculation, OD_600_ measurements were obtained as previously described.

For these experiments, peak OD_600_ was obtained for each biological/technical replicate by averaging a window of seven time points around the maximal value before 36 h. One replicate for the 120 µM concentration had an anomalous shape compared to the other five replicates, and its peak OD_600_ was therefore excluded. Peak OD_600_ values were then used to fit a standard linear model, and means and 95% confidence intervals were obtained using the emmeans R package (91). Plots were made using the ggplot2 (102) R package.

### Growth with riboflavin degradation products

To evaluate whether potential degradation products of riboflavin could support growth, we supplemented the chemically defined medium with ribose, ribitol, or lumichrome at a final concentration of 200 µM. Because of its low solubility in water, lumichrome stock solutions were prepared in DMSO, so we additionally performed a DMSO vehicle control. Growth curves were performed under strict anaerobic conditions inside the Coy anaerobic chamber using a microplate protocol, inoculation strategy, and incubation conditions as described above. Optical density at 600 nm (OD_600_) was measured using an Alto plate reader (Cerillo) without shaking.

### Growth in Balch/Hungate tubes and measurement of hydrogen production

To evaluate the growth under 100% CO_2_, growth experiments were conducted using Balch and Hungate tubes outside the anaerobic chamber. Chemically defined media were prepared and described as above, either with 17.5 µM or 0.13 µM of riboflavin. After preparing the media, 9.5 mL was dispensed into Hungate or Balch tubes. All tubes were sealed and sparged with 100% CO_2_ for 20 min to establish anaerobic conditions and remove residual oxygen. Following gas exchange, tubes were stored at 4°C until use.

*Clostridium scindens* was grown under 100% CO_2_. Three independent colonies were picked from BHI-CHK plates and inoculated into BHI-CHK broth inside the anaerobic chamber, followed by incubation at 37°C for 24 h. After incubation, 2 mL of culture were harvested by centrifugation at 4,000 rpm for 10 min inside the anaerobic chamber. Supernatants were decanted, and cell pellets were resuspended in 10 mL of pre-reduced chemically defined media and transferred into sterile Hungate tubes prior to removal from the chamber.

For inoculation, 0.5 mL of the resuspended culture was transferred into prepared Hungate and Balch tubes using a sterile 1 mL syringe fitted with a 23G needle. Following inoculation, tubes were incubated statically at 37°C in the dark. Growth was monitored by measuring OD_600_ at hourly intervals through the tubes.

Following completion of growth measurements, hydrogen production was quantified from the headspace of the Hungate and Balch tubes using gas chromatography. Headspace samples (250 µL) were collected from each tube using a 1 mL gas-tight syringe and injected into a gas chromatography equipped with a thermal conductivity detector (TCD) and helium as the carrier gas. Hydrogen separation was achieved using a molecular sieve column. Uninoculated medium controls were processed in parallel to account for background hydrogen levels.

### Extracellular sampling and analysis

To quantify and characterize metabolites over time, *C. scindens* was grown in Hungate tubes under anaerobic conditions outside of the Coy anaerobic chamber, using the same media preparation and inoculation strategy, and 100% CO_2_, as described above. Chemically defined media were supplemented with 17.5 µM of riboflavin.

Cultures were inoculated by transferring 500 µL of resuspended cells into Hungate tubes. At different time points following inoculation (0, 0.5, 1, 2, 4, and 6 h), 1.5 mL of cultures were collected from individual tubes. Samples were then centrifuged at 15,000 rpm for 5 min, and the supernatant was collected and immediately snap-frozen for downstream high-performance liquid chromatography (HPLC) analysis. Following sample collection, tubes were discarded and not reused, resulting in destructive sampling each time point. All experiments were performed using three biological replicates. Tubes and supernatants were maintained in the dark, with care taken to minimize light exposure during sampling and measurement to avoid photodegradation of flavins.

Frozen supernatant samples were thawed on ice prior to analysis. For each sample, 200 µL of supernatant was mixed with 300 µL of ultrapure water to reduce viscosity and then transferred into LC vials.

HPLC analysis was performed on a Shimadzu Prominence system with UV detection at 260 nm and 215 nm wavelengths. Compounds were resolved using a MacMod Altima C18 reversed-phase column at 30°C. The mobile phase consisted of water containing 0.025% trifluoroacetic acid (Buffer A) and acetonitrile containing 0.025% trifluoroacetic acid (Buffer B). Separation was carried out at a flow rate of 1 mL/min from 0% B to 45% B over 15 min after an initial hold at 0% B for 5 min. A total volume of 480 µL was injected for each HPLC run.

Riboflavin and lumichrome were quantified by liquid chromatography–mass spectrometry (LC–MS/MS) using a Sciex Triple Quad 3500 mass spectrometer coupled to a Shimadzu HPLC system. Separation was achieved on a reversed-phase C18 column (Synergi Fusion-RP, 80 Å, 4 µm, 50 × 2.0 mm; Phenomenex) maintained at 40°C, with a flow rate of 0.2 mL/min⁻¹ and a sample injection volume of 10 µL. The mobile phase consisted of water containing 0.025% trifluoroacetic acid (solvent A) and acetonitrile containing 0.025% trifluoroacetic acid (solvent B). The gradient program was 0 min, 100% A; 1 min, 5% B; 9 min, 50% B; 10–11 min, 75% B; and 12–14 min, 5% B. Detection was performed using positive electrospray ionization with multiple reaction monitoring. Riboflavin was monitored at m/z 377→243 (declustering potential 65 V; collision energy 32 V) and eluted at 6.3 min, while lumichrome was monitored at m/z 243.2→198 (declustering potential 124 V; collision energy 30 V) and eluted at 7.6 min.

### Fraction collection and identification

To further characterize the compound corresponding to the peak of interest eluting at approximately 23 min, supernatant samples were subjected to preparative fraction collection during HPLC analysis using the same chromatographic conditions as described above. Fractions corresponding to the peak of interest were manually collected between 22.5 min and 23.5 min during the chromatographic run in a 1.5 mL tube. Subsequently, the collected samples were analyzed by LC-MS/MS as above mentioned for compound identification.

### RNA sample collection, extraction, and sequencing

RNA sequencing experiments were conducted using *C. scindens* grown under the same anaerobic conditions and experimental setup described above for the tube-based growth experiments under 100% CO_2_. Cultures were prepared, inoculated, and incubated identically, except that the experiments were performed exclusively in Balch tubes. Cultures were incubated statically at 37°C. Sampling time points were selected to capture transcriptional dynamics across growth phases. Samples were collected at 11, 12, 14, and 24 h following inoculation. At each time point, 5 mL of culture was collected using a sterile syringe fitted with an 18G needle and immediately transferred into 10 mL of RNA Protect reagent to stabilize RNA. Samples were mixed thoroughly and processed according to the manufacturer’s instructions. Total RNA was extracted using the RNeasy Mini Kit following the manufacturer’s protocol. The base media samples at 11 h post-inoculation had very low RNA yields and were excluded from further analysis. Extracted RNA was stored at −80°C until downstream analysis.

RNA was quantified using the Qubit RNA Assay Kit (Invitrogen, Thermo Fisher, Carlsbad, CA, USA), and 425 ng was used for the RapidOut DNA Removal Protocol (Thermo Fisher Scientific, Waltham, MA, USA). Genomic DNA was removed by treating samples with 1 µL of RNase-free DNase I and 2 µL 10× DNase buffer. Incubation and DNase removal were performed according to the manufacturer’s instructions. Purified RNA was used for RNA sequencing as follows. Ribosomal RNA was depleted from the extracted RNA using the Hybridize Probe, rRNA Depletion, and Probe Removal reaction mixtures from the Illumina Stranded Total RNA Prep Kit (Illumina Inc., San Diego, CA, USA). rRNA-depleted samples were purified using a 2.0× Sbeadex SAB bead cleanup (Biosearch Technologies, Hoddesdon, UK) with a single 80% ethanol wash. Following cDNA library construction and indexing, the cDNA libraries were quantified using the Qubit 1× dsDNA High Sensitivity (HS) Assay Kit (Invitrogen, Thermo Fisher, Carlsbad, CA, USA), and the fragment sizes were assessed using a 4150 TapeStation System (Agilent Technologies, Santa Clara, CA, USA). Samples were then pooled in equimolar amounts and sequenced on an Illumina NextSeq 2000 with paired-end 2 × 150 bp reads at 25 million reads for each sample.

Raw sequencing reads were processed using Trimmomatic (103) v0.38. Adapter sequences and low-quality bases were removed using the following parameters: LEADING:3, TRAILING:3, SLIDINGWINDOW:4:15, and MINLEN:50. Reads were trimmed based on when the average quality of the bases dropped below a Phred score of 15, and reads lower than 50 bases after trimming were discarded.

Trimmed reads were aligned with the *Clostridium scindens* reference genome (41) (NCBI RefSeq Assembly: GCF_004295125.1) using Bowtie2 v2.4.1. Alignment output files were written into SAM format. Alignments were sorted based on chromosomal coordinates using Samtools v1.17. Duplicate reads were identified and marked using the MarkDuplicates function in Picard v3.0.0, with the alignment sort order set to “coordinate,” then retained for downstream analysis.

Gene-level read counts were aligned to an annotated reference genome using the featureCounts tool from the subread v2.0.8 package. Reads were aligned to genomic features of type gene using gene identifiers as meta-features. A count table was exported as a text file for downstream analysis.

Differential gene expression analysis was conducted using the R package DESeq2 (104) v1.46.0. One sample with fewer than 1e6 reads was discarded, and genes with zero variance were also dropped. For significance testing, time points were binned into either early (<18 h) or late (>18 h). A model with a two-way interaction, with one factor representing early vs late time points and the other representing supplemented vs base media, was then fit to the count data; genes with either a significant interaction or a significant media main effect (*p*_adj_ ≤ 0.05, log_2_ fold change ≥ 2, Wald test) were retained. Heatmaps were generated with ComplexHeatmap (105) v2.24.1; count data were first transformed using the regularized log (rlog) transformation in DESeq2. For visualization, each gene was normalized by subtracting out the mean value at 12 h in base (0.13 µM) medium, so that the plotted rlog values would reflect changes relative to this time point.

### Comparative genomics across *Lachnospiraceae* and *Bacteroidales*

To expand comparative analyses across the *Lachnospiraceae* family, representative genomes were selected based on genome quality metrics. Only genomes with estimated completeness greater than 95% and contamination less than 5% were retained for downstream analyses. High-quality genomes meeting these criteria were obtained by searching the Genome Taxonomy Database (GTDB v202.0). We used representative genomes to minimize redundancy and ensure phylogenetic coverage across the family.

Annotation with KOfams and generation of presence–absence matrices was performed as in the previous section. In parallel, genomes were annotated using Prokka (106) (with gene calling by Prodigal) (107) to generate predicted protein sequences. The resulting amino acid FASTA files were used as input for orthogroup identification using OrthoFinder v2.5.5 (58) to assess gene family conservation and copy number variants across *Lachnospiraceae* genomes. OrthoFinder was also used to infer a species tree phylogeny via FastTree (108) based on a concatenated MAFFT (109) alignment of single-copy orthologs, and to produce gene trees for orthogroups via DendroBLAST (110).

Specific orthologs of *fin* genes in *Faecalicatena fissicatena* were identified using MMseqs2 (111) v18.8cc5c to conduct a reciprocal-best-hit (“rbh”) search against *C. scindens* with default parameters. To find other orthologous neighborhoods, we used the trees of the orthogroups containing the bifunctional riboflavin kinase/FAD synthetase (*finB*), class I aldolase (*finC*), zinc-dependent alcohol dehydrogenase (*finD*), and sugar-binding transcriptional regulator (*finE*). For each gene family, the smallest monophyletic clade that included both the *C. scindens* and *F. fissicatena* sequences (from the neighborhood identified using MMseqs2) was chosen as a set of orthologs (see Fig. S4).

Using the same genome selection criteria and comparative genomic pipeline as for the *Lachnospiraceae*, a parallel analysis was performed for members of the *Bacteroidales* order. Representative *Bacteroidales* genomes were selected using the same quality threshold and retrieved from GTDB. Genome annotation, KEGG Ortholog assignment, KO presence–absence matrix construction, orthogroup inference, and species tree reconstruction were performed as described above, except that we used OrthoFinder v3.1.0 (112), running the analysis in two rounds (as suggested by its authors) for better scalability.

Trees were plotted using the R package ggtree (113) v3.16.3, and gene neighborhoods were plotted using the R package gggenes (114) v0.6.0.

### Reanalysis of metabolomics data

Metabolomics data were downloaded from the NIH Common Fund’s National Metabolomics Data Repository (NMDR), the Metabolomics Workbench (115) (https://www.metabolomicsworkbench.org). The specific study accession for Han et al. (62) was ST001683 (project ID PR001074, doi:10.21228/M8W11P); the reversed phase positive ion mode “named metabolite” data were used. The Ho et al. (63) study accession was ST002832 (project ID PR001774, available at doi:10.21228/M8DB1F), and the HILIC positive ion mode “named metabolite” data were used.

## Data Availability

RNAseq data have been submitted to NCBI Gene Expression Omnibus (GEO) repository under accession number GSE319166. DNA sequencing data for the *R. peoriensis* genome are available from the NCBI Sequence Read Archive (SRA) under the BioProject PRJNA1076216.

## SUPPLEMENTAL MATERIAL

Fig. S1 jb.00108-26-s0001.pdf Quantitation of riboflavin metabolites by LC-MS/MS.

Fig. S2 jb.00108-26-s0002.pdf Identification of the late HPLC peak as lumichrome.

Fig. S3 jb.00108-26-s0003.pdf Read coverage plots across the fin neighborhood.

Fig. S4 jb.00108-26-s0004.pdf Orthogroup gene trees for finBCDE.

Fig. S5 jb.00108-26-s0005.pdf Diagram showing detected fin gene neighborhoods in *C. scindens*, *M. intestini*, *F. fissicatena*, and *M. hominis*.

Legends for supplemental material jb.00108-26-s0006.docx Legends for Tables S1 to S6 and Fig. S1 to S5.

Tables S1 to S6 jb.00108-26-s0007.xlsx Strain information, KOfams present in strains that grow well vs poorly, representative genomes used for broad-scale comparative genomics, and potentially orthologous fin neighborhoods.

ASM does not own the copyrights to Supplemental Material that may be linked to, or accessed through, an article. The authors have granted ASM a non-exclusive, world-wide license to publish the Supplemental Material files. Please contact the corresponding author directly for reuse.
