# Supplementary material for: Anaerobic riboflavin degradation by human gut Lachnospiraceae
Source: J Bacteriol. 2026 Jul 20;208(8):e00108-26. doi: 10.1128/jb.00108-26 (PMC13492142; doi:10.1128/jb.00108-26)
Supplement: Supplementary file 1 [file jb.00108-26-s0001.pdf]

A

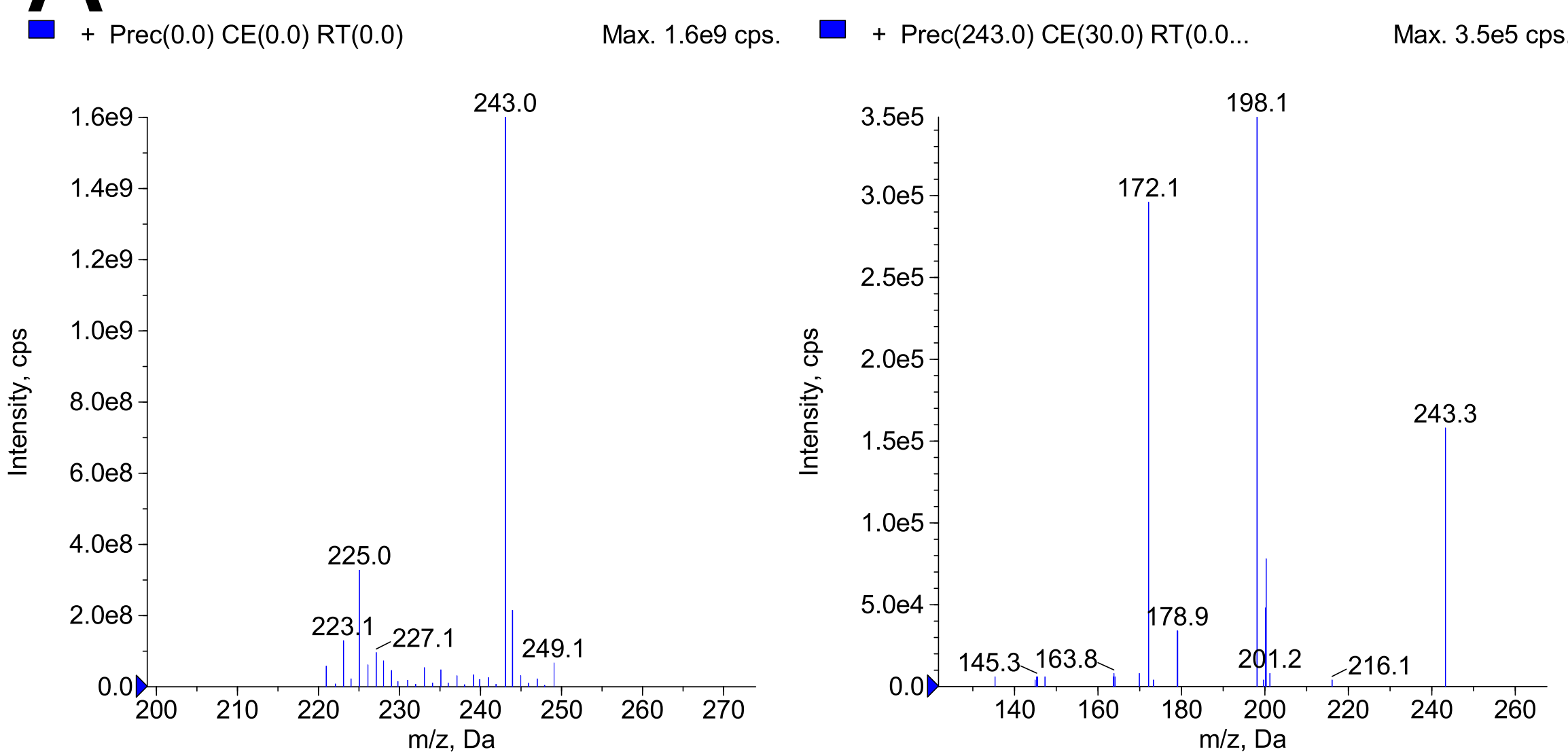
+ Prec(0.0) CE(0.0) RT(0.0)
Max. 1.6e9 cps.
243.0
225.0
223.1
227.1
249.1
Intensity, cps
m/z, Da
+ Prec(243.0) CE(30.0) RT(0.0...
Max. 3.5e5 cps.
198.1
172.1
243.3
178.9
145.3
163.8
201.2
216.1
Intensity, cps
m/z, Da

B

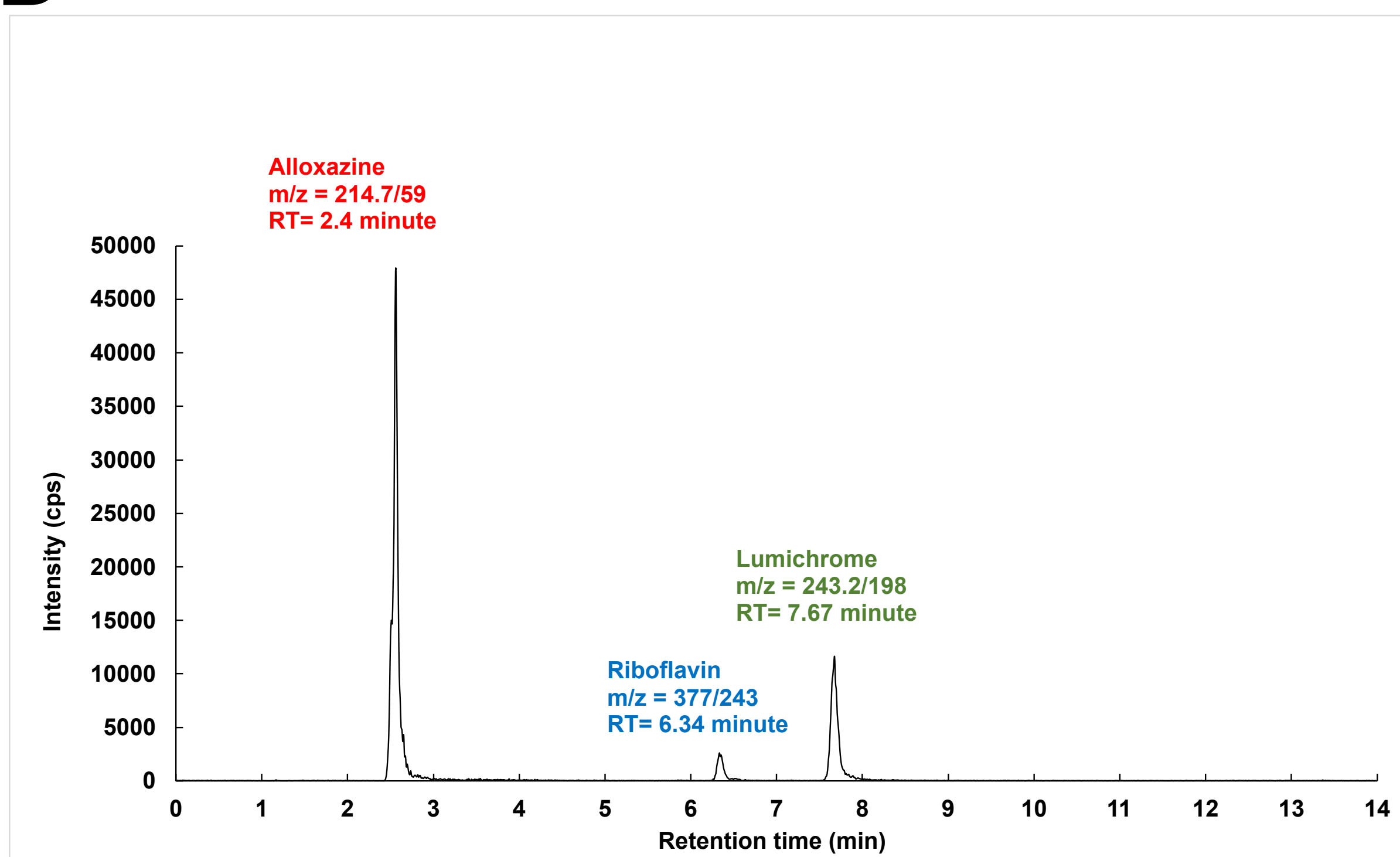
Alloxazine
m/z = 214.7/59
RT= 2.4 minute
Riboflavin
m/z = 377/243
RT= 6.34 minute
Lumichrome
m/z = 243.2/198
RT= 7.67 minute
Intensity (cps)
Retention time (min)
