# Supplementary material for: Anaerobic riboflavin degradation by human gut Lachnospiraceae
Source: J Bacteriol. 2026 Jul 20;208(8):e00108-26. doi: 10.1128/jb.00108-26 (PMC13492142; doi:10.1128/jb.00108-26)
Supplement: Supplementary file 2 [file jb.00108-26-s0002.pdf]

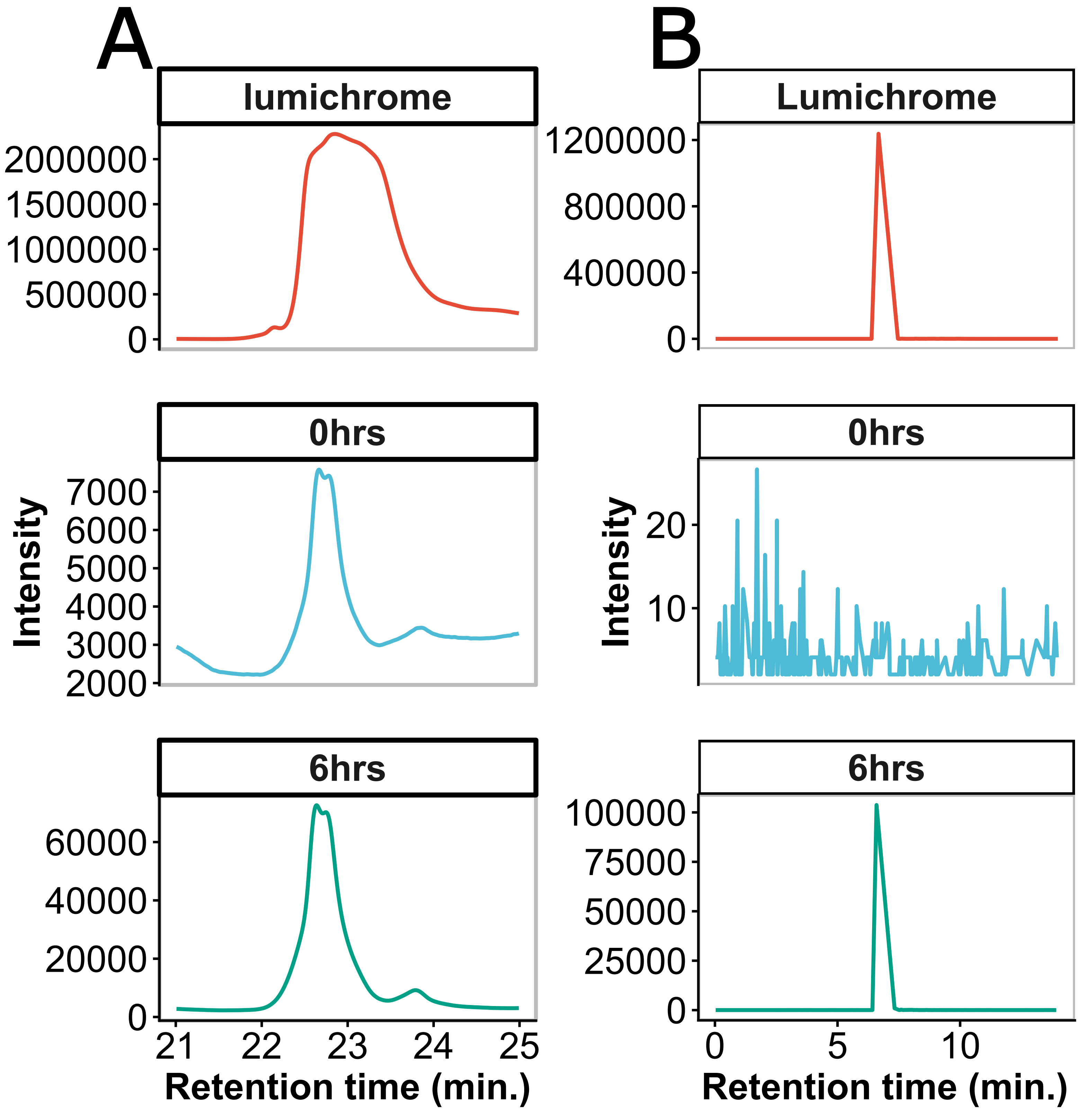
A
B
lumichrome
Lumichrome
0hrs
6hrs
Intensity
Retention time (min.)
2000000
1500000
1000000
500000
0
7000
6000
5000
4000
3000
2000
60000
40000
20000
21
22
23
24
25
1200000
800000
400000
20
10
100000
75000
50000
25000
5
