# Supplementary material for: Anaerobic riboflavin degradation by human gut Lachnospiraceae
Source: J Bacteriol. 2026 Jul 20;208(8):e00108-26. doi: 10.1128/jb.00108-26 (PMC13492142; doi:10.1128/jb.00108-26)
Supplement: Supplementary file 3 [file jb.00108-26-s0003.pdf]

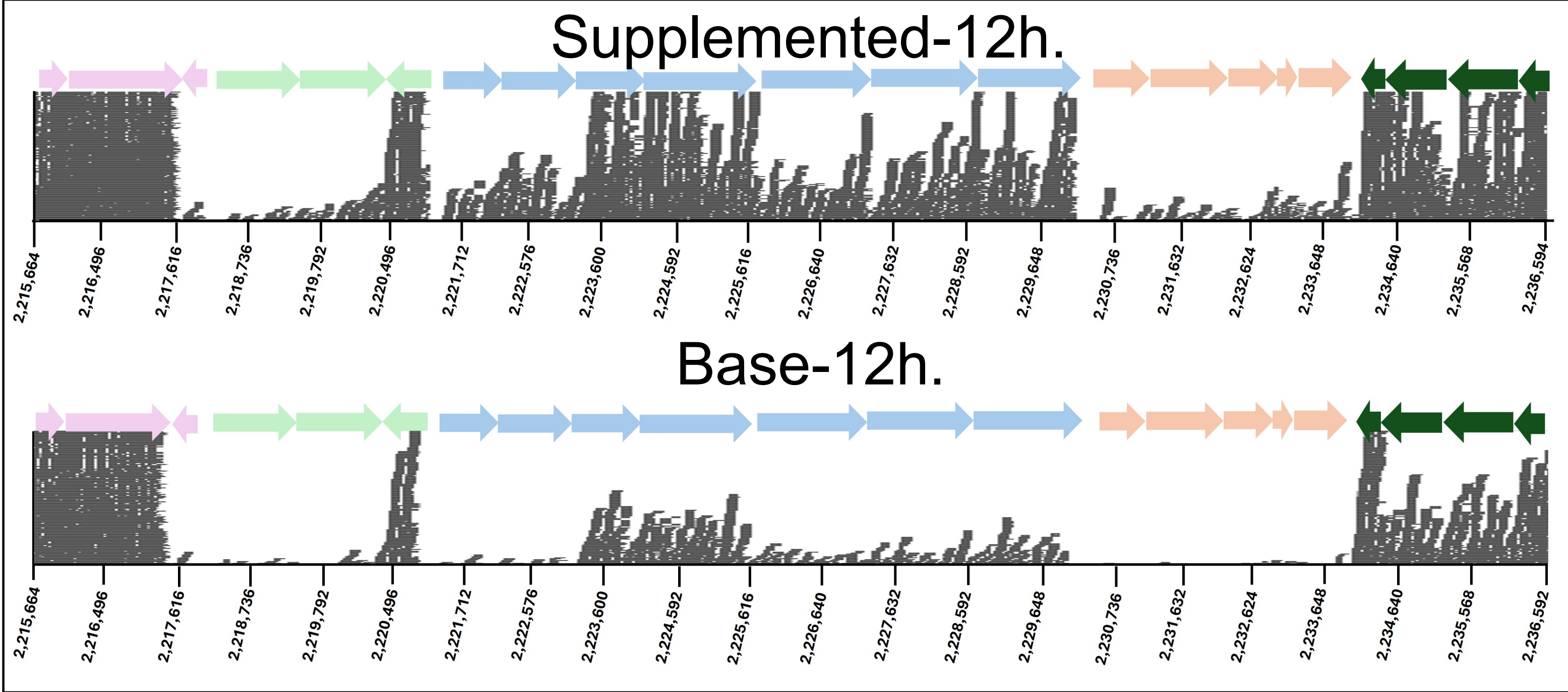
Supplemented-12h.
2,215,664
2,216,496
2,217,616
2,218,736
2,219,792
2,220,496
2,221,712
2,222,576
2,223,600
2,224,592
2,225,616
2,226,640
2,227,632
2,228,592
2,229,648
2,230,736
2,231,632
2,232,624
2,233,648
2,234,640
2,235,568
2,236,594
Base-12h.
2,215,664
2,216,496
2,217,616
2,218,736
2,219,792
2,220,496
2,221,712
2,222,576
2,223,600
2,224,592
2,225,616
2,226,640
2,227,632
2,228,592
2,229,648
2,230,736
2,231,632
2,232,624
2,233,648
2,234,640
2,235,568
2,236,592
