# Supplementary material for: Anaerobic riboflavin degradation by human gut Lachnospiraceae
Source: J Bacteriol. 2026 Jul 20;208(8):e00108-26. doi: 10.1128/jb.00108-26 (PMC13492142; doi:10.1128/jb.00108-26)
Supplement: Supplementary file 4 [file jb.00108-26-s0004.pdf]

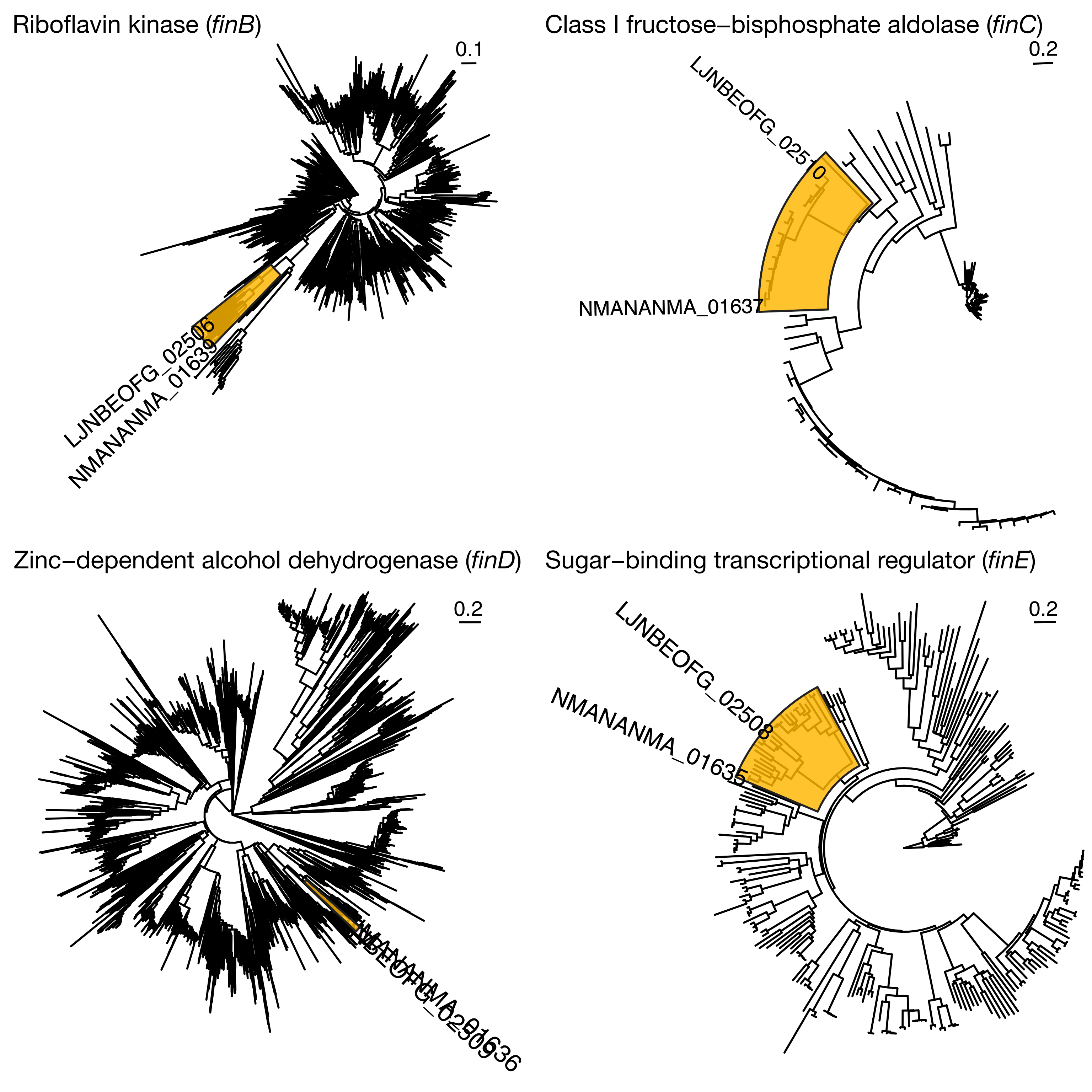

Riboflavin kinase (*finB*)
0.1
LJNBEOFG_02506
NMANANMA_01639
Class I fructose−bisphosphate aldolase (*finC*)
0.2
LJNBEOFG_025
0
NMANANMA_01637
Zinc−dependent alcohol dehydrogenase (*finD*)
0.2
Sugar−binding transcriptional regulator (*finE*)
0.2
LJNBEOFG_02508
NMANANMA_01635
