# Supplementary material for: Anaerobic riboflavin degradation by human gut Lachnospiraceae
Source: J Bacteriol. 2026 Jul 20;208(8):e00108-26. doi: 10.1128/jb.00108-26 (PMC13492142; doi:10.1128/jb.00108-26)
Supplement: Supplementary file 5 [file jb.00108-26-s0005.pdf]

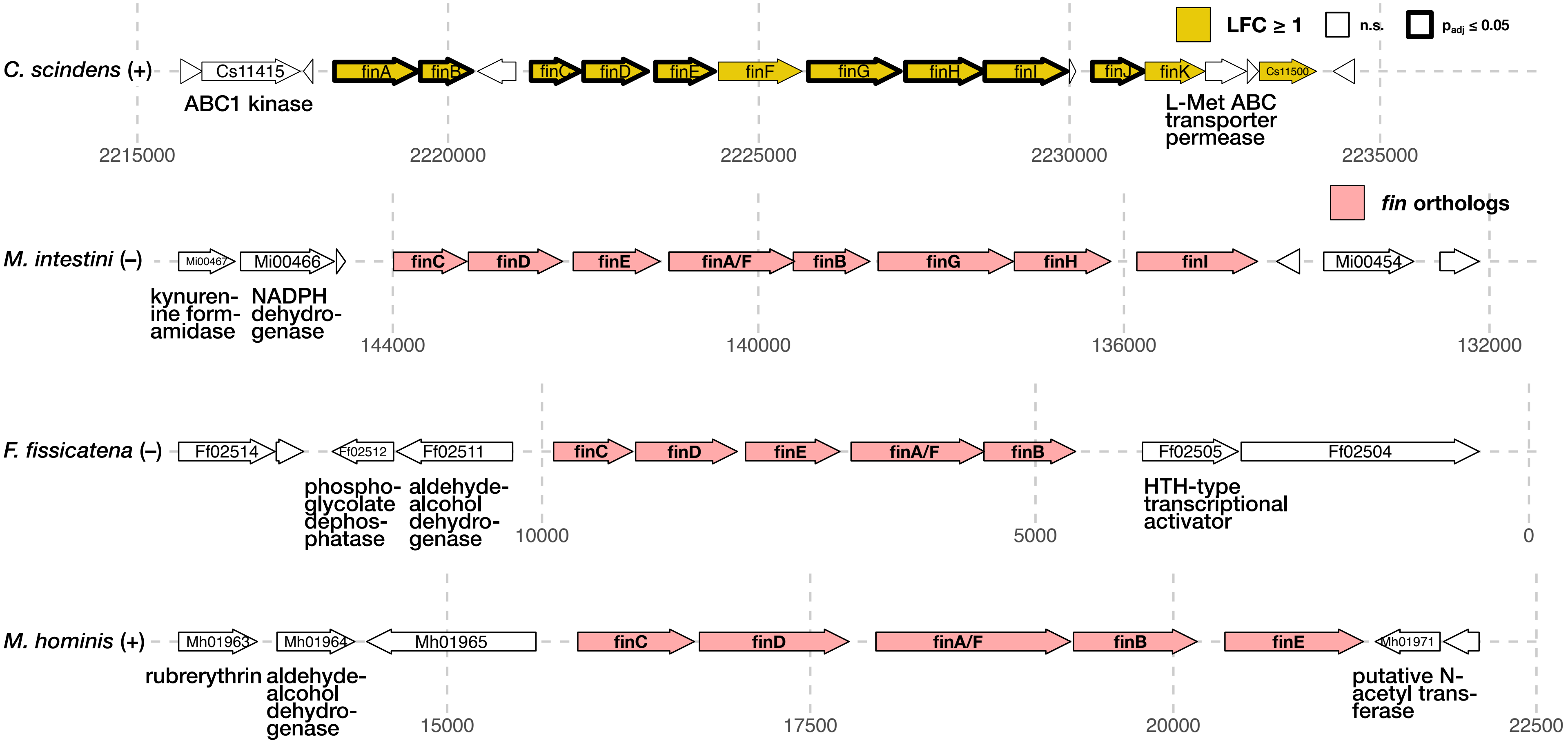

LFC ≥ 1
n.s.
p_{adj} ≤ 0.05
C. scindens (+)
Cs11415
finA
finB
finC
finD
finE
finF
finG
finH
finI
finJ
finK
Cs11500
ABC1 kinase
L-Met ABC transporter permease
2215000
2220000
2225000
2230000
2235000
fin orthologs
M. intestini (–)
Mi00467
Mi00466
finC
finD
finE
finA/F
finB
finG
finH
finI
Mi00454
kynuren-ine form-amidase
NADPH dehydro-genase
144000
140000
136000
132000
F. fissicatena (–)
Ff02514
Ff02512
Ff02511
finC
finD
finE
finA/F
finB
Ff02505
Ff02504
phospho-glycolate dephos-phatase
aldehyde-alcohol dehydro-genase
HTH-type transcriptional activator
10000
5000
0
M. hominis (+)
Mh01963
Mh01964
Mh01965
finC
finD
finA/F
finB
finE
Mh01971
rubrerythrin
aldehyde-alcohol dehydro-genase
putative N-acetyl trans-ferase
15000
17500
20000
22500
