# Supplementary material for: Anaerobic riboflavin degradation by human gut Lachnospiraceae
Source: J Bacteriol. 2026 Jul 20;208(8):e00108-26. doi: 10.1128/jb.00108-26 (PMC13492142; doi:10.1128/jb.00108-26)
Supplement: Supplementary file 6 [file jb.00108-26-s0006.docx]

Supplementary Information

Supplementary Table S1: Detailed strain information for all strains used in this study.

Supplementary Table S2: Table of all KOfams present in *D. formicigenerans*, *R. peoriensis,* and *A. caccae,* but not *C. scindens* or *B. coccoides.*

Supplementary Table S3: Table of all KOfams present in *C. scindens* and *B. coccoides,* but not *D. formicigenerans, R. peoriensis,* or *A. caccae*.

Supplementary Table S4: *Lachnospiraceae* representative genomes used for calling orthogroups.

Supplementary Table S5: Table of all potentially orthologous *fin* neighborhoods detected.

Supplementary Table S6: Bacteroidales representative genomes used for calling orthogroups.

Supplementary Figure S1: Quantitation of riboflavin metabolites by LC-MS/MS. A) Mass spectrum of lumichrome standard before (left) and after fragmentation (right). B) Chromatograms showing elution times for riboflavin, lumichrome, and alloxazine.

Supplementary Figure S2: Identification of the late HPLC peak as lumichrome. A) HPLC chromatograms of (top to bottom) a lumichrome standard, *C. scindens* media pre-inoculation, and *C. scindens* media 6 hrs post-inoculation (note difference in y-axes). B) LC-MS/MS chromatograms showing MRM-based quantitation of lumichrome in HPLC fractions collected directly from the samples in panel A.

Supplementary Figure S3: Read coverage plots across the *fin* neighborhood. A) 17.5µM riboflavin, 11 hours. B) 0.13µM riboflavin, 12 hours.

Supplementary Figure S4: Orthogroup gene trees for *finBCDE*. The *C. scindens* and *F. fissicatena* orthologs are labeled, and the clades selected for identifying potential orthologous neighborhoods are highlighted in orange.

Supplementary Figure S5: Diagram showing detected *fin* gene neighborhoods in *C. scindens, M. intestini, F. fissicatena,* and *M. hominis.* Genes in the same orthogroup as a *C. scindens* *fin* gene are in pink; flanking genes that had a Prokka annotation (other than “hypothetical gene”) are labeled with their inferred function.
